# Effects of immersive virtual reality on limb motor function, balance, gait and quality of life after stroke: A systematic review and meta-analysis

**DOI:** 10.1371/journal.pone.0351114

**Published:** 2026-07-06

**Authors:** Dana Islam, Ingela Marklund, Mia von Euler, Maria Jaensson

**Affiliations:** 1 Örebro University, Faculty of Medicine and Health, School of Health Sciences, Sweden; 2 Centre for Clinical Research and Education, Region Värmland, Sweden; 3 Sophiahemmet University, Stockholm, Sweden; University rehabilitation institute, SLOVENIA

## Abstract

Immersive virtual reality (IVR) has been increasingly integrated into stroke rehabilitation albeit the effectiveness across different outcome domains remains unclear. This study aimed to review evidence from randomized controlled trials evaluating the effects of IVR, used alone or in combination with conventional rehabilitation, on motor-, functional-, and participation-related outcomes after stroke. Ovid MEDLINE, Embase, Cochrane Library and CINAHL were screened up to April 2025 to identify eligible randomized controlled trials (RCTs). A systematic literature search was conducted. Randomized controlled trials including adults with stroke and evaluating immersive virtual reality interventions were eligible. Studies using non-immersive VR, non-randomized designs, or not reporting motor, functional, or participation outcomes were excluded. Meta-analyses were performed where data were sufficiently homogeneous; otherwise, outcomes were summarized descriptively. Risk of bias was assessed using standardized appraisal tools, and certainty of evidence was evaluated using the GRADE approach. Out of 3,574, we included 34 RCTs involving post-acute, subacute and chronic stroke survivors, with a total of1200 participants. Meta-analyses showed IVR, most commonly delivered in combination with conventional rehabilitation, to be associated with improvements in upper extremity motor impairment, particularly measured by the Fugl-Meyer Assessment Upper Extremity (FMA-UE). Small but significant effects were also found for selected gait parameters. In contrast, effects on functional performance, balance, and quality of life were inconsistent and could not be robustly quantified. The overall certainty of evidence ranged from low to very low, primarily due to heterogeneity, methodological limitations, and small sample sizes. In conclusion, IVR appears to be a promising and safe supplement to conventional stroke rehabilitation, particularly for improving motor impairment. However, evidence remains limited and inconsistent across functional outcomes. More well-designed, adequately powered trials with standardized interventions and clinically meaningful outcome measures are needed to clarify the role of IVR in stroke rehabilitation. PROSPERO (Registration number: CRD420250639792).

## Introduction

Stroke is the second leading cause of death and the third leading cause of death and disability in the world [1]. Today, more people survive, but many live with permanent disabilities. This affects not only the individual but also society at large with high costs in municipal and regional healthcare [2,3].

Rehabilitation after stroke is essential. Physiotherapy plays a central role in rehabilitation improving functional capacity and increasing participation in daily activities, thereby affecting stroke survivors’ health-related quality of life [4]. A challenge in stroke rehabilitation is to achieve sufficient exercise intensity and frequency. There is need for new technological interventions that can stimulate and encourage patients to actively participate in their own rehabilitation.

Technology, such as virtual reality (VR), has become increasingly important in stroke rehabilitation [5]. Virtual reality (VR) can be described as a technology that creates a simulated environment in which the user experiences the illusion of physical presence through three-dimensional visual displays, sound and interactive elements. This technology enables interaction with digital objects and environments [6]. In today’s terminology, virtual reality encompasses a spectrum of experiences, including related concepts such as augmented reality (AR), where digital content is superimposed on the real world via devices such as smartphones or glasses, and mixed reality (MR), which integrates physical and virtual elements for real-time interaction. An overarching concept is extended reality (XR), which includes virtual reality (VR), augmented reality (AR), and mixed reality (MR) [7,8]. The level of immersion typically classifies VR: immersive VR (IVR) provides complete immersion with stereoscopic graphics, audio, and motion tracking, isolating the user from the physical world; semi-immersive VR offers partial immersion via large screens or projectors where the user is still aware of their surroundings; and non-immersive VR presents virtual environments on standard displays without depth tracking [9,10]. The present review focused on immersive virtual reality interventions (IVR).

Interest in using IVR in stroke rehabilitation has increased markedly over the past decade [11]. Previous systematic reviews investigating the effects of IVR have generally pointed to its potential as a complement to traditional therapy [12,13]. Despite a growing number of studies and published systematic reviews, significant knowledge gaps remain. Several pervious reviews have combined immersive and non-immersive VR interventions, making it difficult to isolate the specific effects of IVR technologies [13–17]. In addition, many reviews have primarily focused on upper extremity rehabilitation [18–22], while the evidence regarding balance, gait, and broader functional outcomes remains more limited and inconsistent. With rapid technological advancements and a substantial number of studies published in the last years, there is time for a focused systematic review investigating IVR interventions after stroke [11,23]. Thus, an updated and focused systematic review specifically investigating IVR interventions after stroke is essential.

Therefore, the aim of this systematic review was to narratively summarize the evidence on the effects of IVR, used either as a standalone intervention or in combination with other rehabilitation interventions on limb motor function, balance, gait, fatigue and quality of life in adults following stroke, and to quantitatively synthesize the evidence through separate meta-analyses for outcomes where sufficient data were available.

## Method

This systematic review is based on a pre-registered protocol in PROSPERO (Registration number: CRD420250639792) Available at: https://www.crd.york.ac.uk/PROSPERO/view/CRD420250639792 (S1 File). Screening followed the PRISMA guidelines [24].

### Databases and search strategy

A systematic literature search was conducted in four electronic databases: Ovid MEDLINE, Embase, Cochrane Library and CINAHL. The search was developed in collaboration with a research librarian, followed guidelines from the Cochrane Handbook for Systematic Reviews of Interventions, and was guided by the PICO framework [25].

We did not use all PICO elements directly in the database searches. Only the Population (people with stroke) and the Intervention (virtual reality) were used as search terms. According to Cochrane recommendations, terms for Comparison and Outcomes were not included as they can narrow the search and may lead to relevant studies being missed [25,26]. Comparisons are already built into randomized controlled trials, and outcomes are often not mentioned in titles or abstracts [27]. Therefore, PICO guided the overall design of the review, while the database searches focused on broad, sensitive concepts to capture as many relevant studies as possible.

The search combined two main concepts: stroke and virtual reality. Controlled vocabulary and free-text terms for stroke (e.g., Stroke, Hemiplegia, “stroke*”, “cerebrovascular accident*”, “hemipares*”) and virtual reality (e.g., Virtual Reality, “virtual realit*”, “VR”, “head mounted display*”, “smart glasses”) were combined using Boolean operators in all databases. The specific search terms and strategies are detailed in (S2 Table). Limits were set to the English language. Research on immersive VR is predominantly disseminated in English-language journals. Previous systematic reviews in this area have similarly relied on English-language evidence, and bibliometric analyses suggest that most high-impact studies are published in English. Therefore, the likelihood of missing influential or methodologically robust non-English studies is considered low. No date restrictions were applied in any database. The search date was April 1, 2025.

For data synthesis, titles and abstracts of eligible articles were collected using Covidence software [28]. Initial calibration was performed jointly by the doctoral student and supervisors; subsequent screening was performed independently by the reviewers, with each reviewer blinded to the other reviewers’ screening decisions. Disagreements were resolved by a third reviewer.

### Inclusion and exclusion criteria

Studies included if they: (1) involved stroke patients aged ≥18 years; (2) used randomized controlled trial design RCTs; and (3) employed immersive virtual reality interventions, either as a standalone or in combination with conventional rehabilitation. In the present review, IVR was defined as interventions delivered through head-mounted display systems or comparable immersive technologies that provided an immersive three-dimensional virtual environment with user interaction and a sense of presence within the virtual space. The classification of IVR was based on the technical descriptions provided in the included studies.

Studies were excluded if they: (1) used only non-immersive VR; (2) used VR for non-rehabilitative purposes; or (3) primarily focused on cognitive functions.

### Outcomes

The outcomes of interest were limb motor function, balance, gait, fatigue, and quality of life. Limb motor function outcomes included measures assessing upper and/or lower limb motor performance. Balance outcomes included static and dynamic balance measures, and gait outcomes included measures of walking performance, such as walking speed and spatiotemporal parameters. Fatigue and quality of life were defined according to the instruments used in the included studies.

For each outcome domain, data were sought for pre- and post-intervention assessments only. Follow-up outcomes were not collected or synthesized. Outcomes that were not prespecified in the review protocol were not considered.

For quantitative synthesis, pre- and post-intervention data were used to calculate change scores. When multiple outcome measures were reported for the same outcome domain, all relevant measures were considered. When quantitative synthesis was not possible due to insufficient or heterogeneous data, results were summarized narratively.

### Data extraction

Data extraction included study characteristics (author, year, country, participant characteristics, stroke phase), intervention details (sample size, dropouts, blinding, IVR and control interventions, duration and frequency and adverse events), and outcomes data (limb motor function, balance, gait, fatigue, and quality of life), including time points and within- and between- group results.

Data was extracted by one reviewer. To ensure accuracy, the extracted data were independently verified by two senior reviewers, each reviewing approximately half of the studies included. Any discrepancies or uncertainties were resolved through discussion and consensus. When data was missing or unclear, the information was recorded as not reported, and no assumptions were made to impute missing data.

### Risk-of-bias assessment

The methodological quality of the included randomized controlled trials (RCTs) was assessed using the Joanna Briggs Institute (JBI) checklist for RCTs [29], where randomization, allocation concealment, blinding, baseline comparability, follow-up and statistical analysis were evaluated.

Three reviewers independently assessed the methodological quality of the included studies. One reviewer assessed all the studies, while the other two each assessed half. Any disagreements were resolved through consensus discussions. The final assessment of the studies was based on questions Q1–Q3 (randomization) and Q4–Q6 (blinding), with studies were categorized as having lower or higher risk of bias based on the predefined assessment criteria.

### Assessment of the certainty of evidence

The certainty of evidence for each outcome was assessed using the Grading of Recommendations, Assessment, Development, and Evaluations (GRADE) methodology [30,31]. RCTs were initially rated as high-certainty evidence and subsequently downgraded based on predefined GRADE domains: risk of bias, inconsistency, indirectness, imprecision, and publication bias.

### Data synthesis and analysis

A combined narrative synthesis and meta-analysis was conducted. Initially, all included studies were summarized narratively to describe study characteristics, interventions, outcome measures, stroke phase, and risk of bias. Prior to quantitative synthesis, study characteristics were tabulated and compared, including intervention type, outcome domain, measurement instruments, and availability of pre–post data. Studies were grouped for synthesis according to outcome domain, and separate meta-analyses were conducted for outcomes of upper limb movement and function, balance, and gait.

Meta-analyses were performed only when at least three studies within each outcome domain reported comparable outcome measures and sufficient quantitative data (sample size, means, and standard deviations), as fewer studies were considered insufficient to provide reliable estimates of between-study heterogeneity in random-effects models [32]. Quantitative analyses were conducted in R (version 4.3.1) [33], using the meta package [34] and the metafor package [35].

Meta-analyses for continuous outcomes (limb motor function, balance, gait) were conducted using change scores (pre-post) and standardized mean differences (Hedges g) with a random-effects model, accounting for expected clinical and methodological heterogeneity [36,37]. To assess the robustness of the assumed pre-post correlation, sensitivity analyses were performed with alternative correlation coefficients (r = 0.3 and r = 0.7), in line with recommendations from Chapters 6 and 10 of the Cochrane Handbook [32], where the number of included studies was sufficient to allow meaningful interpretation. For outcomes with very few included studies, sensitivity analyses were not performed, as such analyses were unlikely to yield reliable or informative results. Statistical heterogeneity was assessed using the I² statistic and was categorized as low if I² < 25%, moderate if I² was between 25 and 50%, and high if I² > 50% [38].

Results of individual studies were tabulated to summarize study characteristics, intervention details, outcome measures, and risk of bias. Quantitative results were visually displayed using forest plots for each meta-analysis. When quantitative synthesis was not possible, results were summarized narratively in the text. The certainty of evidence for each outcome was presented in a Summary of Results table based on the GRADE approach [39].

## Results

### Study selection

**Fig 1:**
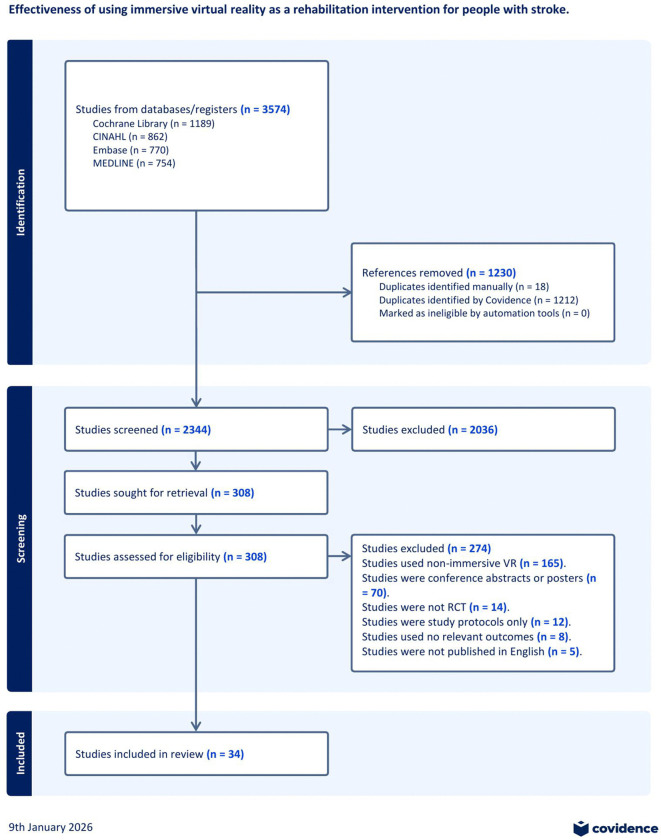
PRISMA 2020 flow diagram of the study selection process.

The database search initially identified a total of 3,574 articles: 754 from MEDLINE, 770 from Embase, 1,189 from the Cochrane Library, and 862 from CINAHL. After removing duplicates, 2,344 articles remained and were independently screened by three reviewers based on titles and abstracts. In total, 34 RCTs published between 2008 and 2024 met the inclusion criteria and were included in the final systematic review (Fig 1). A list of excluded studies with reasons for exclusion is provided in (S3 Table)

### Study characteristics

The 34 RCTs included in the analysis involved 1,224 participants with a stroke. The studies covered different stages of recovery: 21 focused on the chronic phase (>6 months post-stroke) [40–60], 10 on the subacute phase (1–6 months post-stroke) [61–70], and 3 on the acute phase (<1-month post-stroke) [71–73].

The participants’ mean ages ranged from 42 to 68 years. There were more men in the studies, 325 men and 200 women in the intervention groups and 334 men and 214 women in the control groups. Four studies included 3 comparison groups, allowing the researchers to evaluate IVR interventions against multiple types of control conditions [45,47,53,66],

The included studies were conducted across multiple countries, with the largest number of studies coming from Korea (n = 10) [47,48,51–54,56,57,66,71], China (n = 8) [42,45,46,50,59,62,65,68], United States (n = 5) [40,43,61,65,68], and Taiwan (n = 6) [45,46,50,59]. Overall, most studies were conducted in Asia, with smaller contributions from Europe, North America, Africa, and Oceania.

There was an increase in publications after 2020, reflecting both expanding research activity and rapid technological advances in IVR-based stroke rehabilitation.

Additional information about studies and participants are presented in (Table 1).

**Table 1:** Characteristics of the patient.

Number	Study	Participants Total (intervention (n)/ control (n))	Number of withdrawals	Sex (Men/Female) – InterventionControl	Mean Age (SD) – InterventionControl	Intervention	Control	Training	Total IVR-time (hours)	Outcome Measures	JBI
**1**	**Abdollahi 2014** USA	27 (13/14 crossover group).Chronic.	1	12/14 (overall)	57.92 (overall, SD not provided)	IVR + CT.VR Robotic and OpticalOperations Machine (VRROOM)	**Standard:** Repetitive practice without augmentation.	**Intervention:** 60 min/d (45 IVR + 15 min ROM+ setup), 3d/w, 2 w (Then switch). **Control:** 60 min Standard training/d,3d/w, 2w	270 min = 4,5 h	FMA-UE, WMFT, BBT	L-risk of bias
**2**	**Amin 2024** Pakistan; New Zealand; USA	52 (26/ 26). Subacute.	0	16/1018/8	51.8 ± 12.949.8 ± 9.9	VRGI+CPT.Standalone Oculus Quest 2 Virtual Reality device, which uses hand-tracking technology	Conventional Physical Therapy (CPT)	4 d/w, 6 w. During the first 2 weeks (24 min VR + 24 min COT), for the next 4 weeks (40 min VR + 40 min COT)	840 min = 14 h	FMA-UE, ARAT, BBT, SSQOL	H-risk of bias
**3**	**Bao 2024** China	70 (35/ 35). Subacute.	0	19/1618/17	48.27 ± 8.1148.65 ± 8.34	IVR + traditional Chinese medicine health preservation therapy. FundamentalVR	CT + Traditional Chinese medicine healthpreservation therapy	**Intervention:** IVR: 40 min/d, 6 d/w, 4w + traditional Chinese medicine health preservation therapy 3d/w. **Control: CRT:** 50 min 3d/w + traditional Chinese medicine health preservation therapy 3d/w	960 min = 16 h	FMA-UE	H-risk of bias
**4**	**Bashir 2024** Italy and Pakistan	60 (30/ 30).Chronic.	0	16/416/4	59.5 ± 7.260.2 ± 6.9	IVR + PT.The CAREN (Computer-Assisted Rehabilitation Environment) system + PT for balance rehabilitation.	Standard PT program for balance rehabilitation.	4 w. The sources do not specify the frequency of the sessions (e.g., how many sessions per week) or the duration of each individual session for either intervention group		BBS, TUG, FRT	H-risk of bias
**5**	**Chen 2023** China	50 (25/25).Chronic.	22	16/917/8	56.4 ± 12.959.1 ± 10.2	IVR-based exercise to upper limb exercises.HTC Vive Pro, HMD, hand-held controller, laptop computer, and elastic bandage.	IVR-based commercial games	**Intervention:** 35 min/d, 6 d/w, 2 w. **Control:** 35 min/d, 6 d/w, 2 w	420 = 7 h	FMA-UE, WMFT, EQ-5D-5L,	L-risk of bias
**6**	**Connelly 2010** USA	16 (7/7).Chronic.	2	4/34/3	57 ± 1854 ± 10	IVR + GlovePneuGlove, HMD, wide field-of-view (140° vertical, 150° horizontal).	IVR only.The same training without the Glove device.	**Intervention:** 30 min IVR + 30 min training with real objects /d, 3d/w, 6 w. **Control:** 30 min IVR + 30 min training with real objects / d, 3d/w, 6 w	540 min = 9 h	FMA-UE, BBT	H-risk of bias
**7**	**Crosbie 2012** UK	18 (9 / 9).Chronic.	1	5/45/4	56.1 ± 14.564.6 ± 7.4	IVR. HMD, a motion tracking system, and sensors applied to the shoulder, elbow, and hand	Conventional arm therapy.	**Intervention:** 30–45 min/d, 3d/w, 3w. **Control:** 30–45 min/d, 3d/w, 3w.	270-405 min = 4,5–6,5 h	ARAT, UEMI	L-risk of bias
**8**	**Dabrowska 2023** Czech	70 (35/ 35). Subacute.	20	13/1213/12	59.3662,96	IVR + CT.Oculus Quest 2	CT only	**Intervention:** IVR: 20 min/d, 3d/w, 4–5 w (avg. 270 min total) + PT + OT **Control:** PT 30 min/d, 2d/w + OT 30 min/d, 2d/w.	270 min = 4,5 h	BBS	H-risk of bias
**9**	**Hegazy 2022** Saudi Arabia; Egypt	20 (10/10). Subacute.	0	N/AN/A	54.20 ± 3.256.40 ± 3.6	IVR program + task-oriented program.PlayStation console, IVR goggles.	Task-oriented program only.	**Intervention:** IVR: 10–15 min/d, 3d/w, 6 w + 60 min TOT. **Control:** 60 min TOT/d, 3d/w, 6 w	270 min = 4,5 h	UEFi	L-risk of bias
**10**	**Hsu 2022** Taiwan, China	54 (18/18/18).Chronic.	2	12/69/88/9	52.9 ± 11.856.9 ± 13.056.7 ± 11.5	IVR-based MT + TOT.Oculus Rift IVR headset + Leap Motion Controller (LMC).	COT = sensorimotor stimulation and skill training for ADLs.MT = mirror therapy, mirror box.	**VR-MT:** 20 min TOT + 30 min VR-MT/d, 2 d/w, 9 w. **COT:** 20 min TOT + 30 min COT /d, 2 d/w, 9 w. **MT:** 20 min TOT + 30 min MT /d, 2 d/w, 9 w.	540 min = 9 h	FMA-UE, BBT	L-risk of bias
**11**	**Huang 2022** Taiwan, China	30 (15/15).Chronic.	0	9/611/4	50.80 ± 12.3258.33 ± 11.22	IVR)-based motor control training (IVRT) +CT. VR headset by HTC VIVE,HMD device.	Conventional occupational therapy (COT)	**Intervention**: 60 min/d (30 IVR), 2–3 d/w, 4–5 w. **Control:** 60 min/d, 2–3 d/w, 4–5 w.	360 min = 6 h	FMA-UE	L-risk of bias
**12**	**Huang 2024** China; USA	40 (20/ 20). Subacute.	12	13/711/9	63.3 ± 14.365.1 ± 6.1	IVR + conventional rehabilitation.HMD—HTC Vive-VR; wireless controllers.	Conventional rehabilitationonly (PT + OT)	**Intervention:** 60 min/d (30 IVR), 5 d/w, 3 w. **Control:** 60 min/d, 5 d/w, 3 w.	450 min = 7,5	FMA-UE	L-risk of bias
**13**	**Jo 2024** Korea	45 (15/15/15).Chronic.	0	7/88/78/7	51.73 ± 13.6351.00 ± 12.97.47.13 ± 13.91	360 MT + CT.The VR system comprised HMD	TMT + CT. CG: CPT	**360MT:** 30 min IVR/d, 3d/w, 4 w + 60 min/d CT, 5d/w, 4 w. **TMT:** 30 min/d + 60 min/d CT, 5d/w, 4 w, 3d/w, 4 w. **CG:** 60 min/d CT, 5d/w, 4 w.	360 min = 6 h	FMA-UE, MFT, BBT	L-risk of bias
**14**	**Jung 2012** Korea	21 (11/10).Chronic.	0	7/46/4	60.5 ± 8.663.6 ± 5.1	IVR treadmill training. HMD.	Standard treadmill training.	**Intervention:** 30 min/d, 5d/w, 3 w. **Control:** 30 min/d, 5 d/w, 3 w.	450 min = 7,5	TUG, ABC	L-risk of bias
**15**	**Kim 2009** Korea	24 (12/12).Chronic.	–	6/67/5	52.42 ± 10.0951.75 ± 7.09	IVR+ conventional PT. IREX IVR system	Conventional PT	**Intervention:** IVR: 30 min/d + 40 min PT, 4 d/w, 4 w. **Control:** 40 min PT/d, 4 d/w, 4 w	480 min = 8 h	BBS, 10MWT, MMAS	L-risk of bias
**16**	**Kuo 2023** Taiwan, China	37 (19/18).Chronic.	3	13/915/3	57.47 ± 6.9959.50 ± 10.65	IVR + conventional OT.The PABLO Tyromotion device	COT	**Intervention:** 30 min IVR + 30 min OT/d, 2d/w, 9 w. **Control:** 60 min OT/d, 2d/w, 9 w.	540 min = 9 h	FMA-UE, BBT, SIS,	L-risk of bias
**17**	**Kwak 2024** Korea, Germany	44 (18/18).Chronic.	8(4 FIVR, 4 Control)	10/811/7	54.28 ± 17.7459.17 ± 13.86	FIVR + CT.Oculus Quest 2 HMD	General physical therapy CT	**Intervention:** 30 min FIVR/d, 3d/w, 5 w + 30 min CT/d, 5d/w, 5 w.**Control:** 30 min CT/d, 5d/w, 5 w.	450 min = 7,5	BBS, TUG, Gait Step Length, Gait (Stride Length, Gait Velocity	H-risk of bias
**18**	**Kwon 2012** Korea	26 (13/13).Acute.	–	9/45/8	57.15 ± 15.4257.92 ± 12.32	IVR + CT. IREX VR system	CT: PT + OT	**Intervention:** 30 min IVR + 70 min CT/d, 5d/w, 4 w. **Control:** 70 min CT/d, 5d/w, 4 w.	600 = 10 h	FMA-UE, MFT	L-risk of bias
**19**	**Lee CH 2014** Korea	21 (10/11).Chronic.	3	8/26/5	47.9 ± 12.054.0 ± 11.9	IVR + PT. Super Video Graphics Array (SVGA) HMD.	General physical therapy program	**Intervention:** 30 min IVR/d, 3d/w, 4 w + 30 min PT/d, 5 d/w, 4 w. **Control:** 30 min PT/d, 5 d/w, 4 w	360 min = 6 h	TUG, BBS, Gait Step Length, Gait (Stride Length, Gait Velocity, Gait cadence.	L-risk of bias
**20**	**Lee SJ 2014** Korea	64 (20/20/19).Subacute.	5	12/89/1110/9	63.1 ± 10.360.6 ± 14.160.3 ± 11.3	cathodal tDCS + VR therapy. VR system HDM	**VR therapy alone:** VR instead of occupational therap. **Cathodal tDCS:** Cathodal tDCS during occupational therapy	**All 3 groups:** 30 min/d, 5d/w, 3 w + CT.	450 min = 7,5	FMA-UE, MFT, BBT	L-risk of bias
**21**	**Loganathan 2024** India	24 (12/12).Acute.	0	7/59/3	53.08 ± 13.8155.08 ± 15.09	VR + CT.Oculus Quest 2, HDM.	CT: impairment specificexercises.	**Intervention:** 25–30 min IVR + 30 min CT/d, 5d. **Control:** 60 min CT/d, 5d.	150 min = 2,5	CMSA	H-risk of bias
**22**	**Marda 2023** India	32 (16/16). Subacute.	0	N/AN/A	N/AN/A	VR based balance training + CT. VR headset	Task oriented balance training + CT.	**Intervention:** 30 min VR + 15 min CT/d, 3d/w, 4w. **Control:** 30 min TOT + 15 min CT/d, 3d/w, 4w.	360 min = 6 h	BBS, POMA	H-risk of bias
**23**	**Mekbib 2021** China; USA	28 (12/11). Subacute.	5	9/38/3	52.17 ± 13.2661.00 ± 7.69	VR + OT. HTC Vive tracking stations. HMD. Leap Motion tracking technology.	OT alone	**Intervention:** 60 min VR + 60 min OT/d, 4d/w, 2 w. **Control:** 120 min OT/d, 4d/w, 2w	480 min = 8 h	FMA-UE	L-risk of bias
**24**	**Moon 2024** Korea	60 (18/19 /17).Chronic.	6(2 VRT, 1 VR, 3 TPT)	10/811/810/7	57.72 ± 11.2860.47 ± 11.4257.82 ± 10.26	VRT: VR + TPT.Oculus Quest 2, a standalone VR headset.	VR group only. Traditional PT: Bobath, PNF for flexibility/strength/movement.	**Intervention: VRT** 30 min VR + 30 min TPT/d, 2d/w, 8 w. **Control: VR only:** 60 min VR/d, 2d/w,8 w. **TPT:** 60 min TPT/d, 2d/w,8 w	480 min = 8 h	BBS, TUG, ABC, 10MWT	H-risk of bias
**25**	**Ogun 2019** Turkey	65 (33/32).Chronic.	19	28/523/9	61.48 ± 10.9259.75 ± 8.07	IVR only. HMD. Leap Motion	CT + sham VR.	**Intervention:** 60 min VR/d, 3d/w, 6 w.**Control:** 45 min CT + 15 min sham VR/d, 3d/w, 6w	1080 min = 18 h	FMA-UE, ARAT	L-risk of bias
**26**	**Park 2013** Korea	16 (8/8).Chronic.	0	6/25/3	46.25 ± 6.8448.75 ± 8.81	VR + CPT. HMD	CPT: lower extremity strengthening, balance/gait training. + CPT.	**Intervention:** 30 min VR/d, 3d/w, 4 w + 60 min CPT/d,5d/w, 4w. **Control:** 60 min CPT/d, 5d/w, 4 w.	360 min = 6 h	Gait Step Length, Gait (Stride Length, Gait Velocity, Gait cadence, 10 MWT	H-risk of bias
**27**	**Pelaez-Velez 2023** Spain	26 (12 /12). Subacute.	2	9/37/5	51.91 ± 18.5859.58 ± 15.97	VR + NPT.Oculus Quest 2 + Kinect + Blexer software.	Neurological physiotherapy NPT	**Intervention:** 15 min VR + 60 min NPT/d, 3d/w, 6 w. **Control:** 60 min NPT/d, 3d/w, 6 w	270 min = 4,5 h	BBS, Tinetti balance, Tinetti Gait.	L-risk of bias
**28**	**Shaphe 2018** Saudi Arabia	28 (14/14).Chronic.	5	N/AN/A	42.7 ± 11.346.5 ± 13.4	VR + PT.Closed loop visual use HMD + treadmill training.	Traditional PT training.	**Intervention:** 60 min PT/d, 6d/w, 4 w + alternate day VR (30 VR).**Control:** 60 min PT/d, 6d/w, 4 w		SF-SIS, Gait Stride Length, Gait Velocity, Gait cadence.	H-risk of bias
**29**	**Shin 2015** Korea	35 (16/ 16).Chronic.	3	11/513/3	53.3 ± 11.854.6 ± 13.4	VR + COT. RehabMaster™ system	Conventional OT (Occupational therapy)	**Intervention:** 30 min VR + 30 min OT/d, 5 d/w, 4 w.**Control:** 60 min OT/d, 5d/w, 4 w.	600 min = 10 h	FMA-UE, SF-36	L-risk of bias
**30**	**Sip 2023** Poland	20 (10/10). Subacute.	–	N/AN/A	54.9 ± 3.9859.2 ± 4.34	VR.Oculus Quest 2 VR glasses module.	Classic mirror therapy using reflection.	**Intervention:** 30 min VR/d, 6 d/w, 3 w.**Control:** 30 min MT/d, 6 d/w, 3 w	540 min = 9 h	FMA-UE, SF-36	H-risk of bias
**31**	**Song 2021** Korea	10 (5/ 5).Chronic.	2	3/23/2	64.20 ± 7.0860.00 ± 10.88	Immersive VR-based bilateralarm training (VRBAT). DK2 Oculus Rift and Oculus Rift controller.	Normal bilateral arm training (NBAT)	**Intervention:** 30 min/d, 5d/w, 4 w. **Control:** 30 min/d, 5d/w, 4 w.	600 min = 10 h	MFT	H-risk of bias
**32**	**Subramanian 2013** Canada	32 (16/16).Chronic.	7(3 VE, 4 PE)	12/411/5	62.0 ± 9.760.0 ± 11.0	IVR.3D IVR environment (3D VE).Stereoscopic glasses. Computer Assisted Rehabilitation Environment (CAREN) simulated a supermarket scene.	Physicalenvironment (PE) training.	**Intervention:** 45 min/d, 3d/w, 4 w. **Control:** 45 min/d, 3d/w, 4 w	540 min = 9 h	FMA-UE, WMFT	L-risk of bias
**33**	**Yang 2008** Taiwan, China	24 (11/9).Chronic.	4	5/65/4	55.45 ± 12.1560.89 ± 9.25	IVR-based treadmill training.3D acceleration graphic card, and 3D auditory outputs	Treadmill training	**Intervention**: 20 min/d, 3d/w, 3 w. **Control:** 20 min/d, 3d/w, 3 w.	180 min = 3 h	ABC, Community Walk Test, Walking Speed, WAQ.	L-risk of bias
**34**	**Zakharov 2020** Russian	62 (35/ 27).Acute	–	18/1714/13	68.1 ± 1.665.4 ± 1.9	IVR + CT. ReviVR: HMD + pneumatic cuffs on feet.	Standard rehabilitation (medication, physiotherapy, NMES).	**Intervention:** 15 min IVR/d, 10 d (2.5 hours total).	150 min = 2,5 h	FMA-LE, BBS	H-risk of bias

**IVR:** Immersive virtual reality. **VR:** Virtual Reality, **CT**: Conventional therapy**, OT:** Occupational therapy, **PT:** Physical therapy, **CG:** Control Group**, 360MTG:** 360° immersive virtual reality-based mirror therapy. **TMTG:** traditional mirror therapy. **FIVR:** Full immersive virtual reality**, HMD:** Head-mounted display. **TMT:** Traditional mirror therapy. **FMA-UE:** Fugl-Meyer Assessment Upper Extremity. **FMA-LE:** Fugl-Meyer Assessment Lowe Extremity, **BBS:** Berg Balance Scale. **BBT:** Box and Block Test. **MFT:** Manual function test. **TUG**: Time up & Go, **ABC:** Activities-specific Balance Confidence Scale**, 10MWT:** 10-meter walk test, **MMAS:** Modified Motor Assessment Scale**, SIS:** Stroke Impact Scale, **CMSA:** Chedoke–McMaster Stroke Assessment**, POMA:** Performance-Oriented Mobility Assessment, **ARAT:** Action Research Arm Test, **SF-36:** Short Form Health Survey, **WAQ:** Walking Ability Questionnaire**, UEFi:** Upper Extremity Functional Index. **WMFT:** Wolf Motor Function Test**, UEMI:** Upper Extremity Motor Index**. FRT:** Functional Reach Test**, SSQOL:** Stroke-Specific Quality of Life Scale. **JBI:** Joanna Briggs Institute**, L:** low risk of bias**. H:** High risk of bias

### Intervention characteristics

All included studies used IVR, but the level of immersion and technical details were often unclearly reported. The majority used standalone Head-Mounted Display (HMDs). Many of the studies (26 out of 34) combined IVR training with some form of conventional therapy (e.g., physical therapy or occupational therapy).

**Fig 2:**
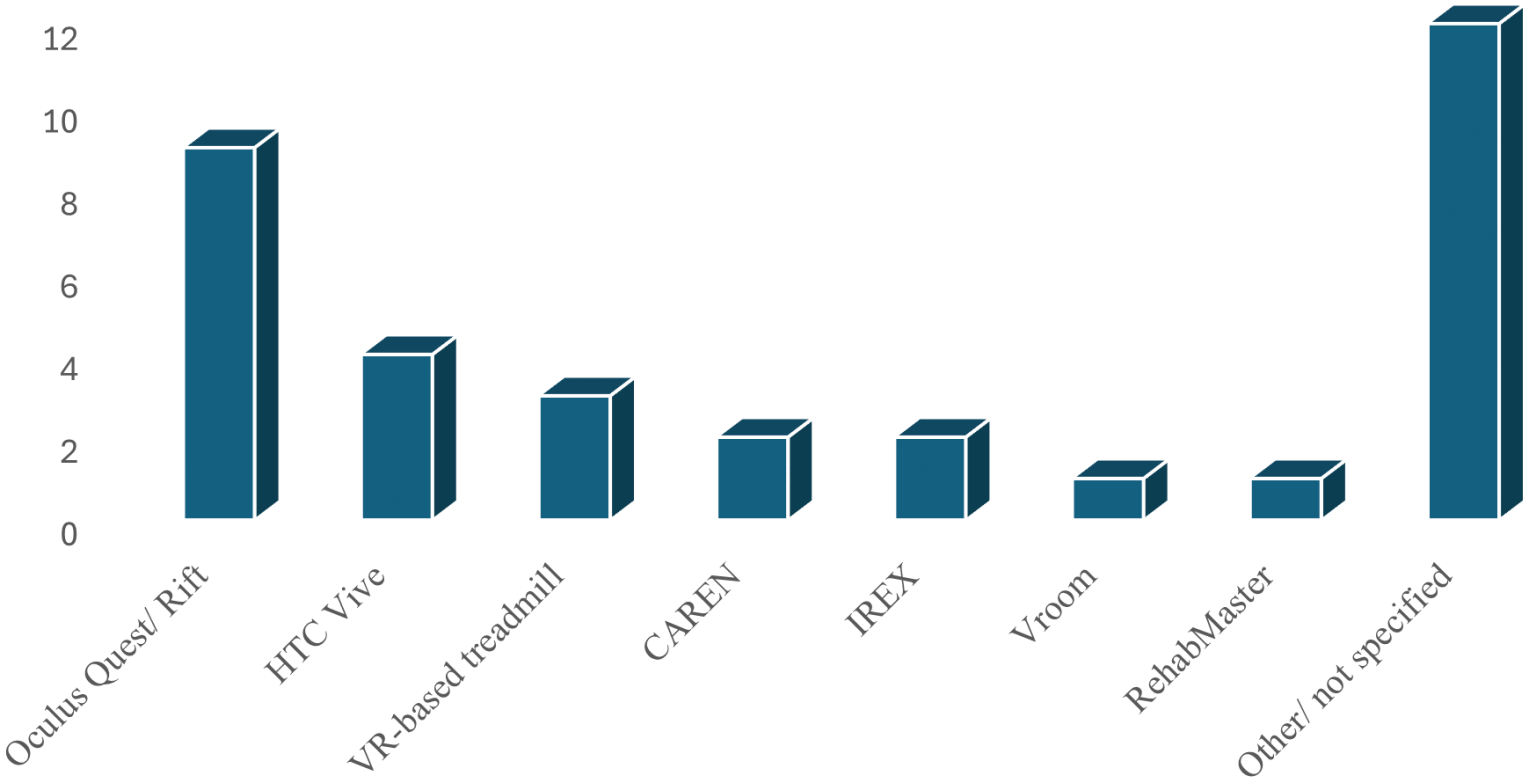
IVR technologies used in the included rehabilitation studies.

The included studies used a spectrum of IVR platforms, from commercial consumer products such as Oculus Quest 2/Rift [45,51,53,57,61,63,69,70,72] and HTC Vive [42,46,65,68] to specialized rehabilitation equipment such as the CAREN (Computer-Assisted Rehabilitation Environment) [41,58] and the RehabMaster system [56] (Fig 2).

The interventions included can be categorized into several main types. First, there is IVR-based upper extremity training, including motor control training, hand tasks, and functional activities. A second major category includes IVR-based gait and balance training, often with simulated environments or treadmill integration. A third category consists of combined interventions that combine IVR with conventional therapy, mirror therapy, or task-oriented training.

Training duration and frequency varied considerably between studies. Some studies used only 10–15 minutes of daily IVR training delivered in addition to 60 minutes of conventional therapy, while others applied 30–60 minutes of daily IVR training. Training periods ranged from 2 weeks to 9 weeks, with most studies conducted over a 3–6-week period. This resulted in a cumulative pure IVR exposure of approximately 2.5–18 hours. Standard dosing (30–45 minutes of pure IVR per session, 3–5 times/week, 4–6 weeks; total 6–10 hours of pure IVR) was seen in most studies.

Patient position varied according to rehabilitation goal: sitting was dominant for upper-extremity tasks [60] and standing or a walking belt was used for balance and gait training [48,51,59].

Control groups received conventional physiotherapy or occupational therapy, standard rehabilitation games, traditional mirror therapy, or treadmill training, typically with intervention durations comparable to or longer than the IVR sessions.

### Outcomes measures

The outcomes were categorized into upper limb movement and function, balance, gait, and quality of life (QoL). These outcomes were assessed by comparing results before and after the interventions.

Fatigue, motivation, intervention compliance, and attrition were prespecified outcomes in the review protocol. However, these outcomes were not reported in the included studies and could thus not be extracted or synthesized. Significant findings were more frequently observed when IVR was used as a supplement to conventional therapy.

### Outcomes of upper limb movement and function

Upper extremity movement and function were assessed in 21 studies. Outcomes were grouped into measures of motor impairment, upper limb functional performance, and manual dexterity and hand function.

### Upper extremity motor impairment

Upper extremity motor function was measured with the Fugl–Meyer Assessment for Upper Extremity (FMA-UE). FMA-UE was the most common outcome measure in 17 studies with a total of 728 participants [40,42,43,45–47,50,56,58,60–62,65,66,68,70,71], with most studies including individuals in the chronic or subacute phase after stroke. In most studies, IVR was delivered as a supplement to conventional rehabilitation, while a smaller number of studies used IVR as a stand-alone intervention (Table 1).

Nine studies (53%) reported a benefit for IVR [40,46,47,60–62,65,66,68], while 8 studies (47%) found no differences between groups [42,43,45,50,51,56,58,70]. Significant findings were more frequently observed in studies where IVR was combined with conventional rehabilitation. Among these 9 significant studies, 5 studies were in the chronic phase (56%) [40,46,47,60,68] and 4 in the subacute phase (44%) [61,62,65,66]. Seven studies were assessed as low risk of bias (78%) [40,46,47,60,65,66,68] and 2 studies as high risk of bias (22%) [61,62].

**Fig 3:**
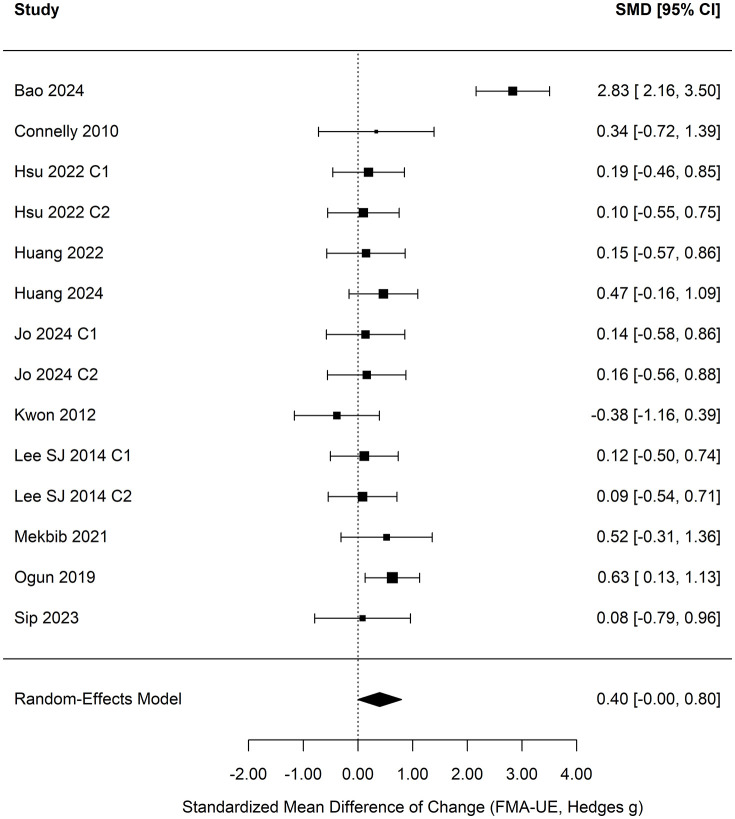
Forest plot of the effect of IVR on upper extremity motor function measured by the FMA-UE (References: [43,45–47,60,62,65,66,68,70,71]).

There was sufficient quantitative data to be included in the meta-analysis from 11 studies [43,45–47,60,62,65,66,68,70,71]. The meta-analysis demonstrated a small to moderate pooled effect in favor of IVR (Hedges’ g = 0.40; 95% CI: 0.00 to 0.80) (Fig 3). The result was borderline statistically significant (p = 0.05). Heterogeneity between studies was large (I² = 78%).

A sensitivity analysis showed that statistical significance was sensitive to the assumed correlation value (r). With a conservative assumption (r = 0.3) the effect was non-significant (Hedges’ g = 0.35; p = 0.058), while an optimistic assumption (r = 0.7) resulted in a statistically significant effect (Hedges’ g = 0.49; p = 0.043).

### Upper extremity functional performance

Upper extremity functional performance was measured with the Wolf Motor Function Test (WMFT) and the Manual Function Test (MFT).

The Wolf Motor Function Test (WMFT) was reported in 3 studies with a total of 109 participants [40,42,58], primarily in individuals in the chronic phase after stroke (Table 1).

One study (33%) reported a benefit for IVR [40], while 2 studies found (67%) no differences between groups [42,58]. All studies were assessed as low risk of bias.

The Manual Function Test (MFT) was evaluated in 4 studies with a total of 145 participants [47,57,66,71], primarily in individuals in the chronic phase after stroke (Table 1).

Two studies (50%) reported a benefit for IVR [47,66], while 2 studies (50%) found no differences between groups [57,71]. Of the studies reporting benefit one was in the chronic [47] and one in the subacute phase [66] and both were assessed as low risk of bias.

**Fig 4:**
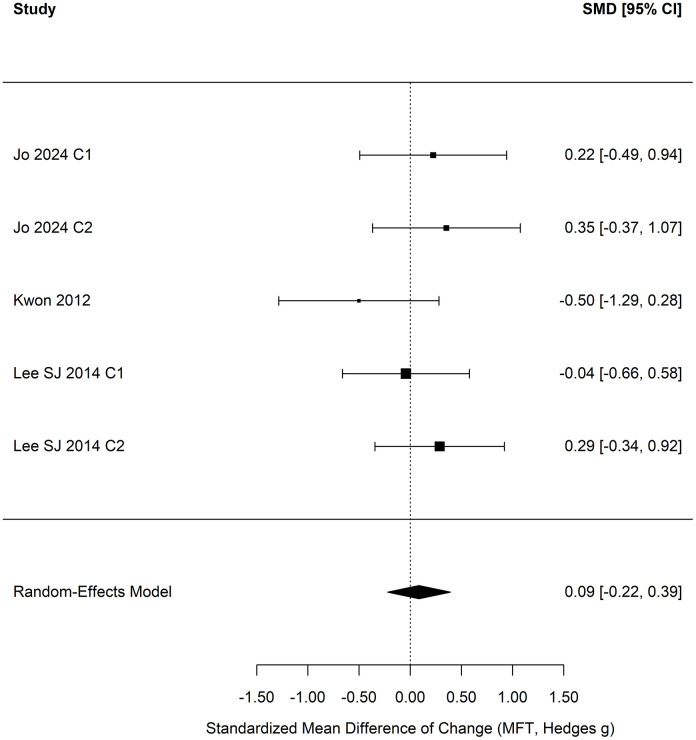
Forest plot of the effect of IVR on upper extremity functional measured by the MFT (References: [47,66,71]).

There was sufficient quantitative data to be included in the meta-analysis in 3 studies [47,66,71]. The meta-analysis showed a non-significant pooled effect (Hedges’ g = 0.09; 95% CI: −0.22 to 0.39) (Fig 4), with no observed heterogeneity (I² = 0%). Sensitivity analysis confirmed that the effect remained non-significant regardless of the assumed correlation value.

### Manual dexterity and hand function

Manual dexterity and hand functional use of the hand and arm were assessed with the Action Research Arm Test (ARAT) and the Box and Block Test (BBT).

The Action Research Arm Test (ARAT) was reported in 3 studies with a total of 135 participants [44,60,61], primarily in individuals in the chronic phase after stroke (Table 1).

Two studies reported a benefit of IVR (67%) [60,61], while 1 study (33%) found no differences between groups [44]. Among the positive studies, 1 was in the chronic [60] and 1 in the subacute phase [61]. One study was assessed as low risk of bias [60] and 1 as high risk of bias [61].

**Fig 5:**
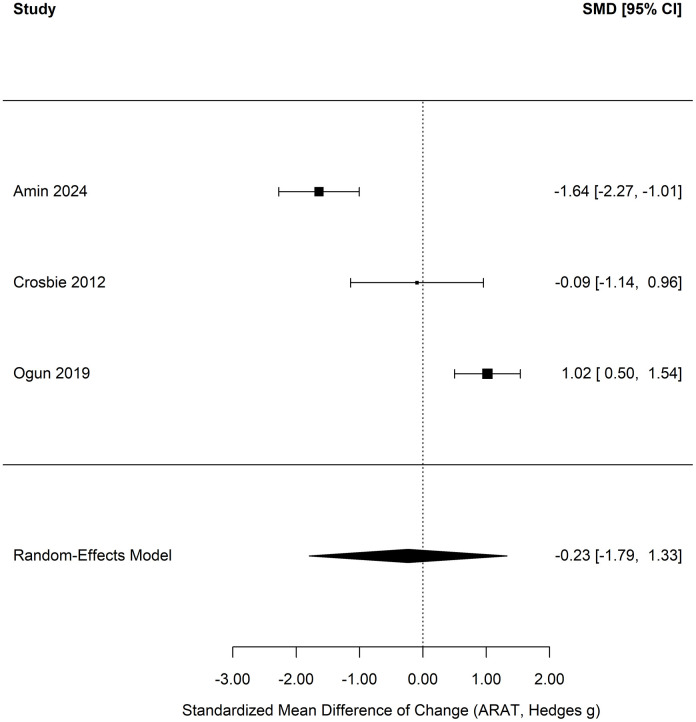
Forest plot of the effect of IVR on manual dexterity and hand functional measured by the ARAT (References: [44,60,61]).

All studies provided sufficient quantitative data for inclusion in the meta-analysis. The meta-analysis showed a non-significant pooled effect (Hedges g = −0.23, 95% CI −1.79 to 1.33). (Fig 5). Heterogeneity between studies was large (I² = 93.37%). Sensitivity analyses were not performed due to the small number of studies included.

The Box and Block Test (BBT) was evaluated in 7 studies with a total of 295 participants [40,43,45,47,50,61,66], primarily in individuals in the chronic phase after stroke (Table 1).

**Fig 6:**
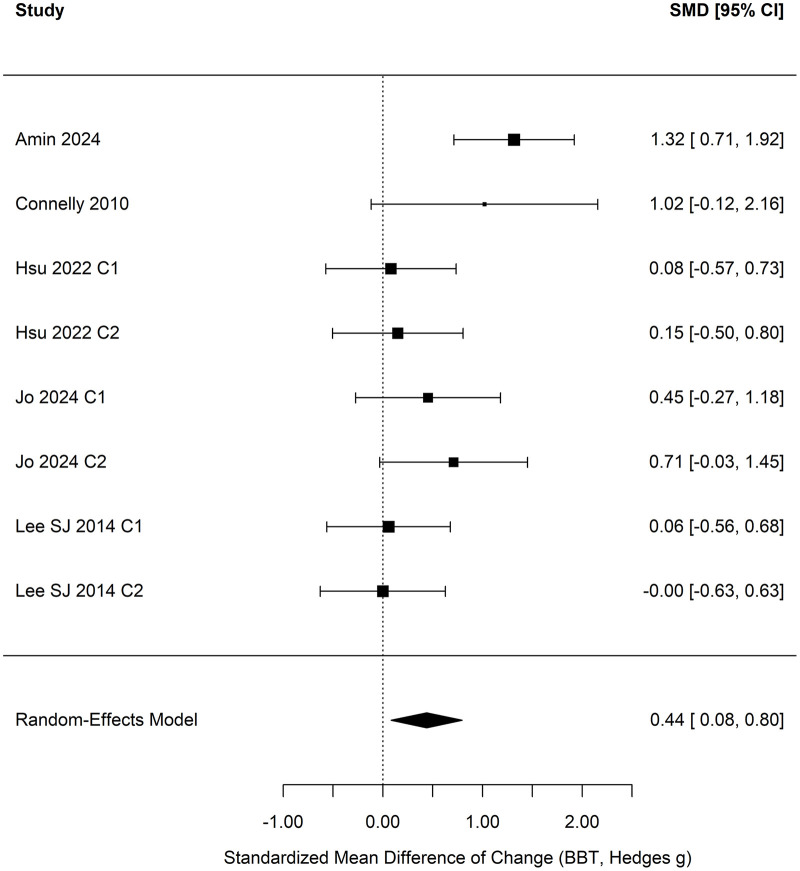
Forest plot of the effect of IVR on manual dexterity and hand functional measured by the BBT (References: [43,45,47,61,66]).

There was sufficient quantitative data to include in the meta-analysis from 5 studies [43,45,47,61,66]. The meta-analysis demonstrated a small to moderate but statistically significant pooled effect in favor of IVR (Hedges’ g = 0.44; 95% CI: 0.08 to 0.80) (Fig 6). Heterogeneity between studies was large (I² = 52.5%). Sensitivity analyses confirmed the stability of the pooled effect across different assumptions of the pre–post correlation. At r = 0.3, the effect was Hedges’ g = 0.38 (I² = 40.4%), and at r = 0.7, the effect was Hedges’ g = 0.55 (I² = 66.4%).

### Outcomes of balance

Balance was assessed in 11 studies. This was primarily assessed using the Berg Balance Scale (BBS), Timed Up and Go (TUG), and Activities-specific Balance Confidence Scale (ABC).

The Berg Balance Scale (BBS) was reported in 9 studies with a total of 399 participants [41,49,51–53,63,67,69,73]. Most studies included participants in the chronic phase after stroke (Table 1).

Five studies reported a benefit of IVR (56%) [49,53,67,69,73], while 4 (44%) found no differences between groups [41,51,52,63]. Of the studies that showed positive results, 2 were conducted in the chronic [49,53], 2 in the subacute [67,69], and 1 in the acute phase [73], 2 of these were assessed as low risk of bias [49,69] and 3 as high risk [53,67,73].

**Fig 7:**
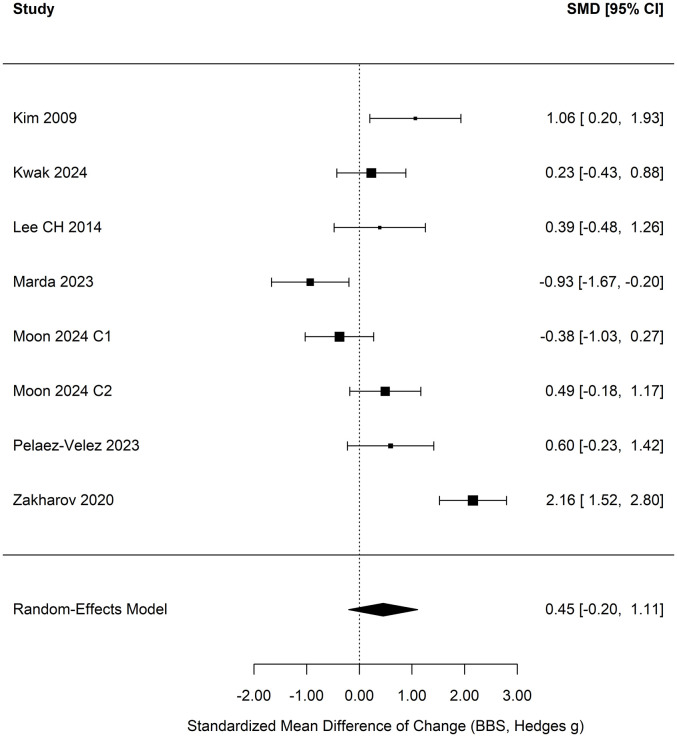
Forest plot of the effect of IVR on balance measured by the BBS (References: [49,51–53,67,69,73]).

There was sufficient quantitative data to be included in the meta-analysis in 7 studies [49,51–53,67,69,73]. The meta-analysis showed no statistically significant pooled effect in favor of IVR (Hedges’ g = 0,45; 95% CI: −0,20 till 1,11) (Fig 7). Heterogeneity was significant (I² = 84.6%). Sensitivity analysis confirmed that the result remained non-significant regardless of the assumed correlation value. At r = 0.3, the effect was Hedges’ g = 0.43 (p = 0.17), and at r = 0.7 the effect was Hedges’ g = 0.54 (p = 0.17).

Timed Up and Go (TUG) was reported in 5 studies with a total of 206 participants [41,48,51–53], with all studies included patients in the chronic phase (Table 1).

Three studies (60%) reported a benefit for IVR [48,51,53], while 2 studies (40%) found no differences between groups [41,52]. Of the studies that showed positive results, 1 was assessed as a low risk of bias [48] and 2 as high risk [51,53].

**Fig 8:**
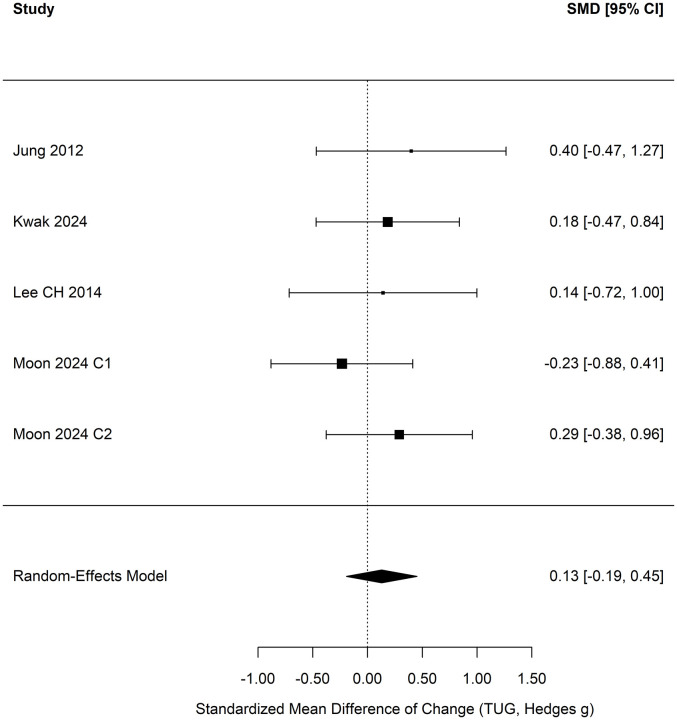
Forest plot of the effect of IVR on balance measured by the TUG (References: [48,51–53]).

There was sufficient quantitative data to include in the meta-analysis from 4 studies [48,51–53]. The meta-analysis did not show a statistically significant pooled effect of IVR compared with control interventions (Hedges’ g = 0,13; 95% CI: −0,19 till 0,45) (Fig 8). The pooled effect estimate was small, with no observed heterogeneity (I² = 0%). Sensitivity analyses with alternative assumptions of pre- and post-correlation yielded consistent results.

The Activities-specific Balance Confidence Scale (ABC) was reported in 3 studies with a total of 105 participants [48,53,59], with all studies including patients in the chronic phase (Table 1).

Two studies reported a benefit for IVR (67%) [48,53], while 1 study (33%) found no differences between groups [59]. Of the studies that showed positive results, 1 was assessed as low risk of bias [48] and 1 as high risk [53].

**Fig 9:**
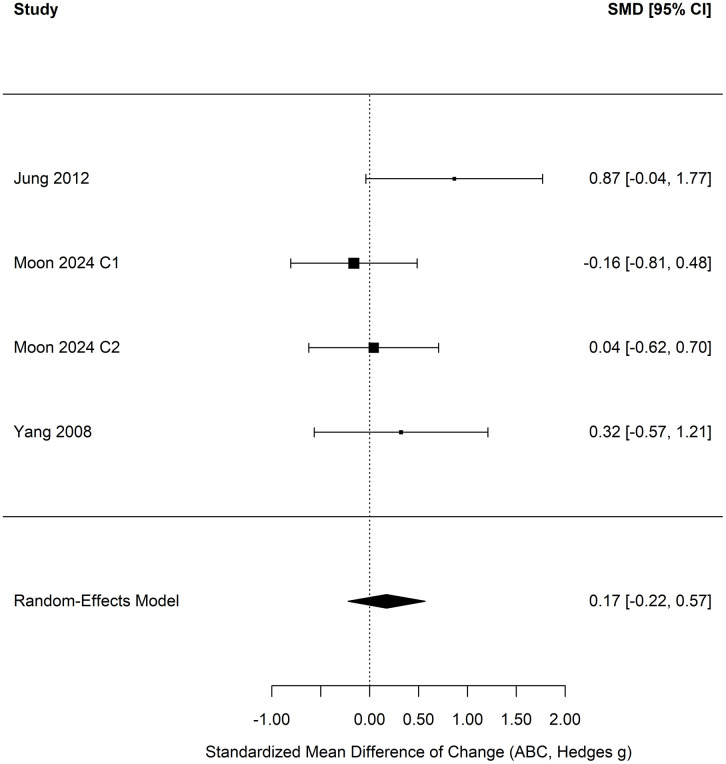
Forest plot of the effect of IVR on balance measured by the ABC (References: [48,53,59]).

All studies provided sufficient quantitative data to be included in the meta-analysis. The meta-analysis showed a small, non-significant pooled effect in favor of IVR, with wide confidence intervals (Hedges g = 0.17, 95% CI −0.22 to 0.57) and low heterogeneity (I² = 8.8%) (Fig 9). Sensitivity analyses were not emphasized for ABC due to the small number of comparisons included.

### Outcomes of gait

Eight studies evaluated gait outcomes using tools such as gait velocity, stride length, step length, cadence and 10-meter walk test (10MWT).

Gait velocity was reported in 4 studies with a total of 109 participants [51,52,54,55], with all studies included patients in the chronic phase (Table 1).

Three studies reported a benefit for IVR (75%) [51,52,55], while 1 (25%) found no differences between groups [54]. Of the studies that showed positive results, 1 of these was assessed as low risk of bias [52] and 2 as high risk [51,55].

**Fig 10:**
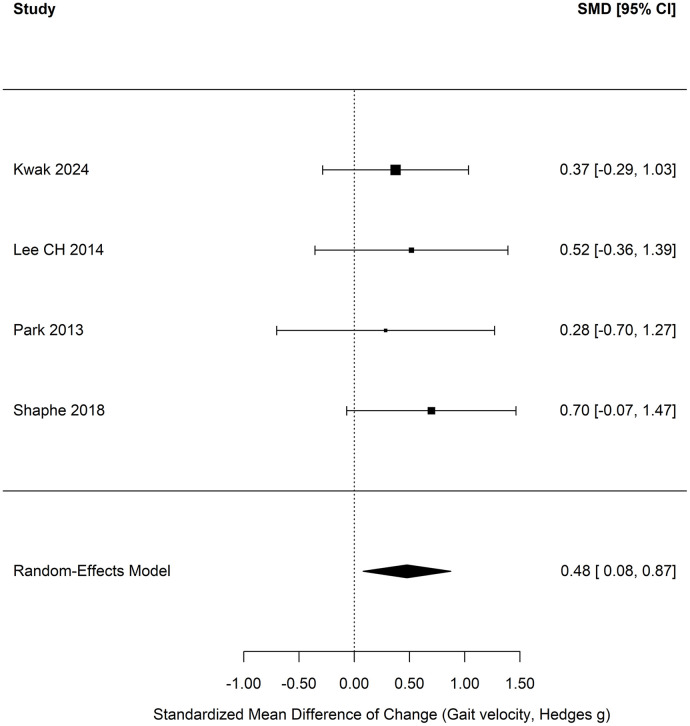
Forest plot of the effect of IVR on gait measured by the Gait Velocity (References: [51,52,54,55]).

All studies were included in the meta-analysis. The meta-analysis demonstrated a statistically significant small to moderate pooled effect in favor of IVR (Hedges’ g = 0,48; 95% CI: 0,08 till 0,87) (Fig 10), with no observed heterogeneity (I² = 0%). Sensitivity analyses using alternative assumptions for the pre–post correlation yielded consistent results.

Step length was reported in 4 studies with a total of 105 participants [49,51,52,54], with all studies including patients in the chronic phase (Table 1).

In a descriptive synthesis, 3 studies reported a benefit for IVR (75%) [49,51,52], while 1 study (25%) found no differences between groups [54]. Of the studies that showed positive results, 2 were assessed as low risk of bias [49,52] and 1 as high risk [51].

**Fig 11:**
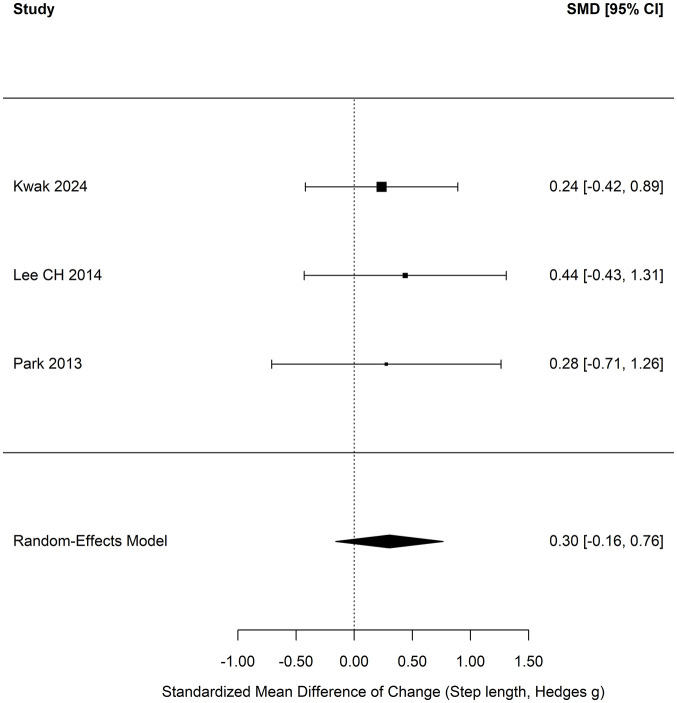
Forest plot of the effect of IVR on gait measured by the Step length (References: [51,52,54]).

There was sufficient quantitative data to be included in the meta-analysis in 3 studies [51,52,54]. The meta-analysis showed no statistically significant pooled effect (Hedges g = 0.30; 95% CI −0.16 to 0.76) (Fig 11)*.* No heterogeneity was observed (I² = 0%). Sensitivity analysis was not performed due to the low number of included studies.

Stride length was reported in 5 studies with a total of 133 participants [49,51,52,54,55], with all studies including patients in the chronic phase (Table 1).

Three studies reported a benefits for IVR (60%) [51,52,55], while 2 studies (40%) found no differences between groups [49,54]. Of the studies that showed positive results, 1 was assessed as low risk of bias [52] and 2 as high risk [51,55].

**Fig 12:**
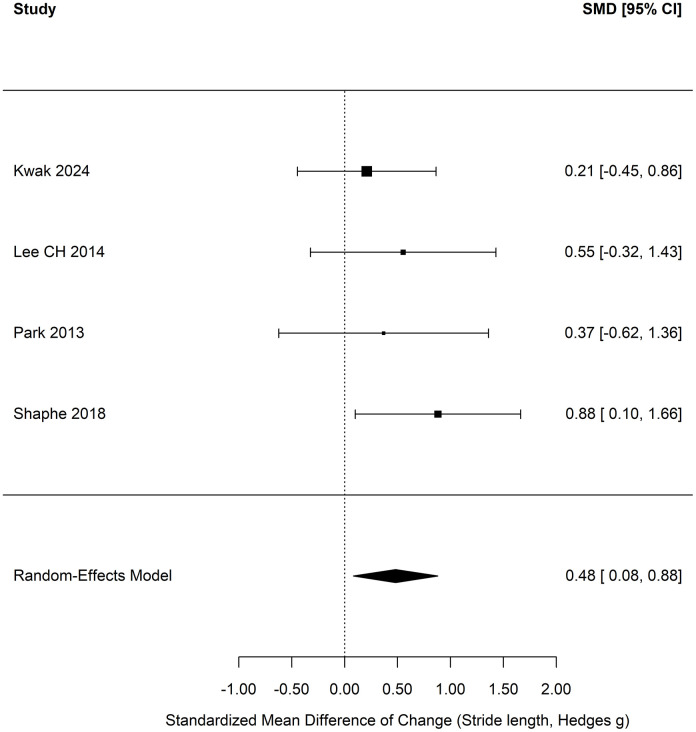
Forest plot of the effect of IVR on gait measured by the Stride length (References: [51, 52, 54, 55]).

There was sufficient quantitative data to include in the meta-analysis from 4 studies [51,52,54,55]. The meta-analysis demonstrated a statistically significant small to moderate pooled effect in favor of IVR (Hedges g = 0.48, 95% CI 0.08 to 0.88**)** (Fig 12), with no observed heterogeneity (I² = 0%).

Cadence was reported in 4 studies with a total of 89 participants [49,52,54,55], with all studies included patients in the chronic phase (Table 1).

Two studies reported a benefit for IVR (50%) [49,55], while 2 studies (50%) found no differences between groups [52,54]. Among the studies that showed positive results, 1 was assessed as low risk of bias [49] and 1 as high risk [55].

**Fig 13:**
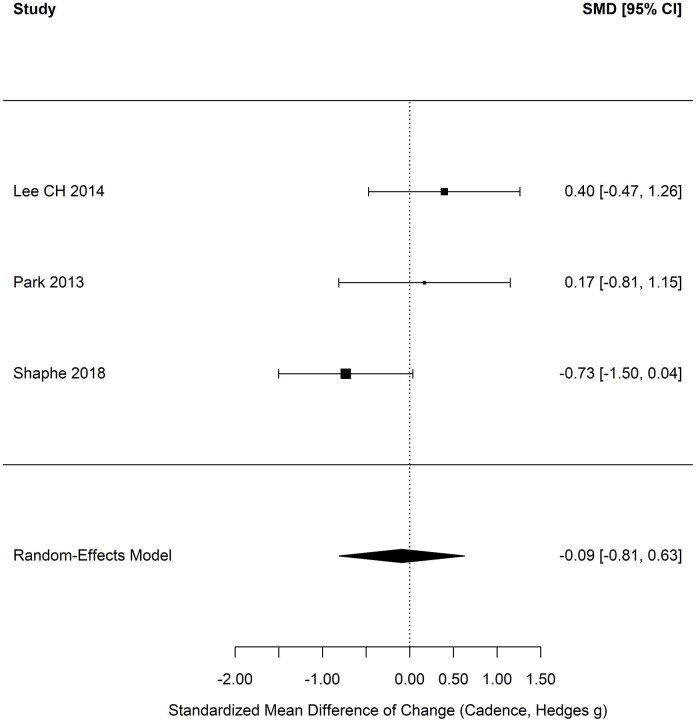
Forest plot of the effect of IVR on gait measured by the Cadence (References: [52, 54, 55]).

There was sufficient quantitative data to include in the meta-analysis from 3 studies [52,54,55]. The meta-analysis showed no statistically significant pooled effect (Hedges g = −0.09, 95% CI −0.81 to 0.63) (Fig 13), and high heterogeneity (I² = 51,45%). Sensitivity analyses were not performed due to the small number of studies included.

The 10-meter walk test (10MWT) was reported in 3 studies with a total of 100 participants [49,53,54], with all studies including patients in the chronic phase (Table 1).

Two studies reported a benefit for IVR (67%) [49,53], while 1 study (33%) found no differences between groups [54]. Among the studies that showed positive results, 1 was assessed as low risk of bias [49] and 1 as high risk [53].

**Fig 14:**
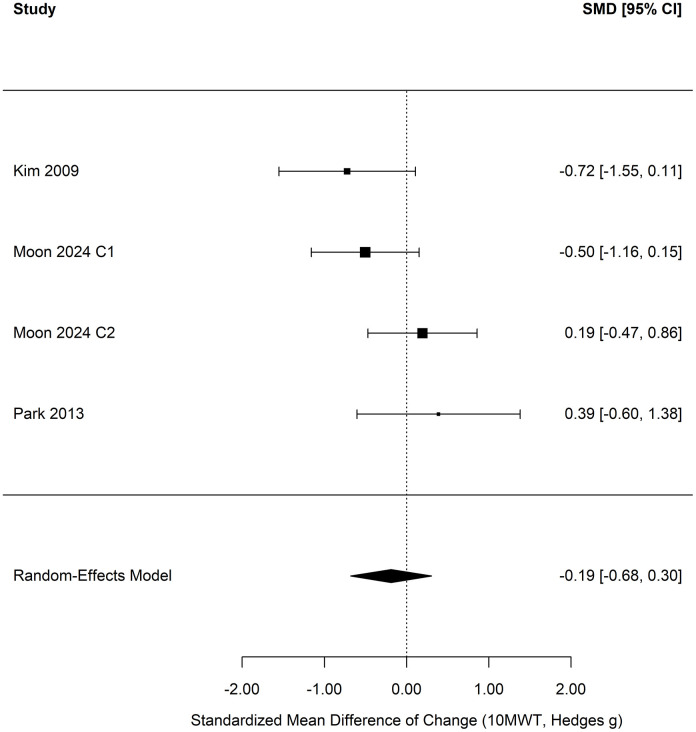
Forest plot of the effect of IVR on gait measured by the 10MWT (References: [49,53]).

All studies were included in the meta-analysis. The meta-analysis showed no statistically significant pooled effect of IVR (Hedges g = −0.19, 95% CI −0.68 to 0.30) (Fig 14). Heterogeneity was low (I² = 39.5%).

### Outcomes of quality of life (QOL)

Quality of life was assessed in 7 studies using instruments such as the Stroke Impact Scale (SIS) and the Short Form Health Survey (SF-36) [74,75].

The Short Form Health Survey (SF-36) was reported in 2 studies with a total of 55 participants [56,70] (Table 1).

One study reported a benefit for IVR (50%) [70], while the other only showed significant improvements in the role limitations due to physical problems domain, but not in other SF-36 domains [56].

The Stroke Impact Scale (SIS) was reported in 2 studies with a total of 65 participants [50,55] (Table 1).

One study reported a benefit for IVR (50%) [55], while the other (50%) found no differences between groups [50]. The study that reported positive results was assessed as high risk of bias [55].

The limited number of studies and variability in outcome reporting precluded quantitative pooling, and the certainty of evidence for quality-of-life outcomes was therefore low.

### Side effects

Side effects were reported in 7 studies (20%) [42,44–46,50,54,76], mostly mild (dizziness, fatigue in <10% of participants), and no serious events were observed, suggesting that IVR is a safe and well-tolerated intervention for individuals with stroke.

Security measures were primarily manual and limited to physical restraints, staff restraints, or seated positions [60,76]. There were no references to modern features such as guardian/chaperone systems, i.e., user-defined virtual safety barriers displayed in real time to protect the user from collisions with the physical environment, which are a standard in all modern consumer headsets [77,78]. This shows that many researchers still use cautious protocols even with modern headsets.

### Methodological quality (JBI Critical Appraisal)

JBI critical appraisal indicates moderate quality, with strengths in basic design and analysis, but significant weaknesses in blinding and allocation. Participant and therapist blinding was rarely feasible due to the nature of rehabilitation interventions involving immersive VR systems. In addition, allocation concealment was unclear or not reported in several studies, which may have increased the risk of performance and selection bias. Outcome assessor blinding was more commonly reported but remained inconsistent across studies.

**Fig 15:**
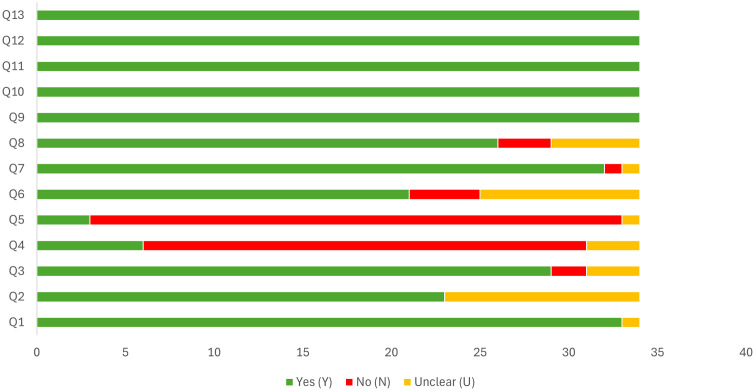
Summary of JBI Checklist results by criterion.

Most studies showed strong methodological consistency across multiple areas. Specifically, true randomization was reported in 33 of 34 studies and equality between groups at baseline in 29 studies, indicating a generally robust study design (Fig 15). Detailed results of the JBI critical appraisal for all included studies are presented in (S4 Table).

Almost all studies reported identical treatment across groups, except for the intervention (32/34), and analyzed participants within their randomized groups (34/34). In addition, outcome measures were applied consistently (34/34) and measured reliably (39/39) across studies. All studies used appropriate statistical analyses (34/34) and appropriate study designs (34/34). These results suggest that the included RCTs maintained high internal validity in terms of randomization, outcome measurement, and data analysis.

### Assessment of Certainty of Evidence (GRADE)

The overall certainty of evidence was classified as low, or very low and summarized in a (Table 2).

**Table 2:** Summary of Findings (SoF) table based on GRADE assessments.

Outcome	No. of studies (participants)	Pooled effect (SMD, 95% CI)	Certainty of evidence (GRADE)	Comments
Upper extremity motor function (FMA-UE)	11 studies (446 participants)	Small–moderate pooled effect. Result was borderline statistically significant.	⊕⊕◯◯ **Low**	Downgraded due to risk of bias and inconsistency (Mixed study results, high heterogeneity in the meta-analysis).
Manual dexterity (BBT)	7 studies (224 participants)	Small pooled effects. Statistically significant effect.	⊕◯◯◯ **Very Low**	Downgraded due to risk of bias, imprecision (Small sample sizes, relatively wide confidence intervals despite significance) and inconsistency (High heterogeneity in the meta-analysis).
Balance (BBS, TUG, ABC)	9 studies (294 participants)	Small pooled effects. No statistically significant.	⊕◯◯◯**Very low**	Downgraded due to risk of bias, inconsistency (Mixed results with substantial heterogeneity in the meta-analysis, primarily for BBS), and imprecision (Small studies, wide CI).
Gait velocity	4 studies (101 participants)	Small–moderate pooled effect. statistically significant effect.	⊕⊕◯◯ **Low**	Downgraded due to risk of bias and imprecision (Limited number of studies and participants, and wide CI).
Cadence	3 studies (65 participants)	Small pooled effects. No statistically significant.	⊕◯◯◯ **Very low**	Downgraded due to risk of bias, inconsistency (High heterogeneity in the meta-analysis) and imprecision (Small number of studies and wide CI).
Step length	3 studies (73 participants)	Small pooled effects. No statistically significant.	⊕⊕◯◯ **Low**	Downgraded due to risk of bias and imprecision (Small number of studies and wide CI).
Stride length	4 studies (101 participants)	No statistically significant pooled effect	⊕⊕◯◯ **Low**	Downgraded due to risk of bias and imprecision (Small number of studies and wide CI).

## Discussion

The central finding of this systematic review and meta-analysis is that IVR, most commonly delivered in combination with conventional rehabilitation, is associated with measurable improvements in selected outcomes, most notably upper extremity motor impairment and certain gait parameters. However, the benefits were not consistent across outcome domains, and the overall certainty of evidence ranged from low to very low. This variability underscores both the potential and the current limitations of our understanding of IVR as a rehabilitation tool.

Unlike several previous reviews that combined immersive and non-immersive VR interventions, the present review focused specifically on immersive VR systems, allowing a more targeted evaluation of immersive rehabilitation approaches after stroke. To our knowledge, this review also includes one of the largest numbers of IVR randomized controlled trials synthesized quantitatively in this field.

Most included studies were conducted in individuals in the chronic phase after stroke, while fewer trials targeted subacute populations. This distribution is consistent with previous reviews [11,20] and may partly reflect practical and ethical considerations, as individuals in the chronic phase in general are more medically stable and easier to recruit [79].

The most robust and consistent effects were observed for upper extremity motor impairments, assessed with the Fugl-Meyer Assessment Upper Extremity (FMA-UE). This finding is consistent with previous systematic reviews [11,12,22] indicating that virtual reality-based interventions tend to show more apparent benefits at the level of impairment than at the level of functional performance. A plausible explanation is that IVR environments are particularly well suited to promote high repetition of goal-directed movements, combined with enhanced visual and sensory feedback – factors considered important drivers of motor relearning after stroke [12,18]. However, the certainty of the evidence was downgraded due to heterogeneity and methodological limitations. Although the pooled effect was small to moderate, direct comparison with established minimal clinically important difference values (MCID) for FMA-UE is limited as MCIDs are defined in scale points rather than standardized effect sizes [32]. The clinical relevance of the observed effects should therefore be interpreted with caution.

In contrast, outcomes assessing upper-limb functional performance, including the Wolf Motor Function Test (WMFT), Manual Function Test (MFT), and Action Research Arm Test (ARAT), showed more variable and generally weaker effects. This pattern has been consistently reported in earlier reviews [12,20] and likely reflects fundamental differences in what these measures capture. While impairment-based assessments primarily assess isolated motor control, functional capacity tests require the integration of multiple motor components under standardized conditions [80,81]. Consequently, improvements at the impairment level may not automatically translate into improved functional performance. As activities of daily living were not prespecified outcomes in the present review, this has not been analyzed.

Manual dexterity, assessed using the Box and Block Test (BBT), demonstrated a small but statistically significant pooled effect favoring IVR. This suggests that immersive environments may be particularly effective for training repetitive, goal-directed hand movements under controlled conditions. The task structure of the Box and Block Test (BBT) closely resembles the interactive and feedback-rich nature of IVR training, which may facilitate engagement and high repetition [82,83]. Nevertheless, as this outcome reflects functional capacity rather than real-world hand use, its clinical implications remain limited.

Balance-related outcomes showed mixed and inconsistent findings. While several trials reported statistically significant improvements in commonly used balance measures, the methodologically robust studies did not confirm these effects. Previous systematic reviews focusing on full IVR [11,84] have reported generally favorable balance outcomes. However, these conclusions were based on narrative syntheses, as substantial heterogeneity in intervention characteristics, outcome measures, and comparator conditions precluded quantitative pooling. Taken together, although IVR appears promising for balance rehabilitation after stroke, the current evidence remains insufficient to draw firm conclusions, and balance-related effects should be interpreted with caution.

For gait-related outcomes, statistically significant pooled effects were identified for gait velocity and stride length, whereas no significant effects were identified for cadence, step length, or walking capacity measured using the 10-meter walk test. Similar patterns, with more consistent effects on gait speed than on other gait parameters and substantial heterogeneity across studies, have been reported in previous reviews [11,16,84]. These findings contrast with the most recent Cochrane review [12], which reported very low-certainty evidence and no clear benefit of virtual reality on gait velocity. This discrepancy may partly be explained by differences in inclusion criteria and analytical focus, as the present review specifically targeted IVR interventions. Nevertheless, despite statistical significance was observed for selected gait parameters, the certainty of evidence for gait outcomes remained low, and many contributing studies were small and judged to be at high risk of bias.

Quality-of-life outcomes were assessed in a limited number of studies using heterogeneous instruments, and the results were inconsistent. This may partly reflect that quality of life is a broad, multidimensional concept influenced not only by physical function, but also by psychological well-being, social participation, expectations, and contextual factors. In this context, the inclusion of activities of daily living outcomes may have provided a more sensitive and clinically relevant indicator of functional transfer, as activities of daily living measures directly reflect an individual’s ability to perform important everyday tasks and are more closely aligned with rehabilitation goals after stroke than quality of life [80,81].

Across outcomes, a defining feature of the included studies was the substantial heterogeneity of IVR interventions. Differences in technology, virtual content, training dose, and mode of delivery complicate direct comparisons and limit conclusions regarding optimal intervention parameters. This challenge has been repeatedly highlighted in previous reviews [11,20–22] and remains a key barrier to evidence synthesis in this field.

### Strengths and limitations of the study

The strength of this review is the comprehensive and systematic search strategy and the inclusion of 34 RCTs all appraised using the JBI Critical Appraisal Checklist. Overall, the included studies demonstrated acceptable methodological quality in key domains such as randomization, baseline comparability, outcome measurement, and statistical analysis, supporting the internal validity of the findings. In addition, sensitivity analyses using alternative pre–post correlation coefficients indicated that the meta-analytic results were robust to key analytical assumptions.

However, several important limitations should be acknowledged. Detailed stroke characteristics such as lesion location, stroke subtype (ischemic versus hemorrhagic), and lesion laterality were inconsistently reported across studies. These factors may influence motor recovery, visual-perceptual function, and responsiveness to IVR interventions, thereby contributing to clinical heterogeneity. Future studies should investigate these variables separately.

Heterogeneity in intervention characteristics, such as differences in IVR systems, training content, session duration, and total intervention dose, likely contributed to variability in treatment effects across studies. In several included studies, IVR was delivered as a supplement to conventional rehabilitation rather than as a direct alternative intervention. Consequently, intervention groups in some studies received greater overall rehabilitation intensity than control groups, which may have contributed to the observed effects independent of the immersive VR component itself.

In addition, most studies involved participants in the chronic phase after stroke, whereas fewer studies targeted acute or subacute populations. As rehabilitation potential may differ substantially across stroke phases, this imbalance may limit the interpretation and generalizability of pooled effects across recovery stages.

Many outcomes were assessed in a limited number of trials with relatively small sample sizes, resulting in imprecise pooled estimates and reduced certainty of evidence, as reflected in the GRADE assessments. Moreover, several outcomes could not be quantitatively synthesized and were therefore summarized narratively, limiting the strength of conclusions for these domains.

Methodological limitations were common. Allocation concealment was reported in only 23 of 34 studies, and blinding of participants and caregivers was rarely implemented, reflecting the inherent challenges of rehabilitation trials involving immersive technologies. Although blinding of outcome assessors and follow-up procedures were better, they remained suboptimal, increasing the risk of selection and performance bias and contributing to the downgrading of evidence certainly.

Furthermore, the classification of immersion level was occasionally challenging due to insufficient technical descriptions of VR systems in some studies, which may have contributed to heterogeneity across studies and the inadvertent omission of relevant trials.

The use of an assumed pre–post correlation coefficient for calculating change score standard deviations represent a methodological limitation. However, sensitivity analyses using alternative correlation values demonstrated that the main findings were robust to this assumption. Nevertheless, many outcomes were based on a small number of studies, limiting the precision of the pooled estimates.

Most of the included studies were conducted in Asian countries, which may affect the external validity of the findings. Variations in healthcare organizations, the intensity of conventional rehabilitation, and access to technology could influence both the implementation and the effects of IVR interventions [85]. Consequently, the transferability of these results to other healthcare settings should be interpreted with caution.

Finally, only studies published in English were included, which may have introduced language bias. However, an additional search without language restrictions identified only two potentially relevant studies (S6 File), suggesting that the impact of this restriction on the overall findings is likely minimal. Additionally, grey literature was not systematically searched. Consequently, potentially relevant unpublished studies or studies published in other languages may not have been identified, increasing the risk of publication bias.

Despite these limitations, this review provides a comprehensive synthesis of the current evidence on IVR in stroke rehabilitation. The generally acceptable methodological quality of the included studies supports the credibility of the finding. Overall, the results suggest that IVR may be a promising supplement to another stroke rehabilitation, particularly for improving motor function and selected gait parameters. At the same time, the observed heterogeneity and limited certainty of evidence highlight the need for larger, well-designed trials with standardized intervention protocols and clinically meaningful outcomes.

### Implications for clinical practice and future research

From a clinical perspective, the findings of this review suggest that IVR may represent a promising supplement to conventional stroke rehabilitation, particularly for improving motor function and selected gait and balance outcomes in the subacute and chronic phases after stroke. Benefits were most consistently observed when IVR was integrated with standard physiotherapy or occupational therapy rather than delivered as a standalone intervention. IVR was generally reported to be safe, with only mild and infrequent adverse effects, supporting its feasibility for clinical use. Still, clinicians should interpret these findings with caution given heterogeneity and the limited certainty of the evidence for several outcomes.

Future research should prioritize adequately powered, well-designed randomized controlled trials with low risk of bias and longer follow-up periods to assess the durability of treatment effects. Greater standardization and clearer reporting of intervention characteristics, including training dose and virtual content, are needed to identify optimal protocols. In addition, future studies should examine whether improvements achieved during IVR training translate into meaningful gains in activities of daily living and participation and include patient-centered outcomes such as quality of life, fatigue, and motivation.

## Conclusion

This systematic review indicates that IVR may represent a promising and safe supplement intervention to conventional stroke rehabilitation, particularly for improving upper extremity motor impairments and selected gait parameters. However, effects were inconsistent across outcome domains, and the overall certainty of evidence ranged from low to very low due to heterogeneity and methodological limitations. Although IVR shows potential, current evidence is insufficient to support firm conclusions about its effectiveness in functional outcomes, balance, and quality of life. Further well-designed, adequately powered studies with standardized intervention protocols and clinically meaningful outcome measures are required to clarify the role of immersive virtual reality in evidence-based stroke rehabilitation.

## Supporting information

S1 File PROSPERO registration for systematic review. (DOCX)

S2 Table Full electronic search strategy. Search strings used for all databases included in the systematic review.
(DOCX)

S3 Table Excluded studies with reasons for exclusion. (XLSX)

S4 Table Risk of bias assessment using the Joanna Briggs Institute (JBI) Critical Appraisal Checklist for Randomized Controlled Trials. (DOCX)

S5 File PRISMA checklist. (DOCX)

S6 File Search without language restrictions. (XLSX)
