# Supplementary material for: Effects of immersive virtual reality on limb motor function, balance, gait and quality of life after stroke: A systematic review and meta-analysis
Source: PLoS One. 2026 Jul 6;21(7):e0351114. doi: 10.1371/journal.pone.0351114 (PMC13336215; doi:10.1371/journal.pone.0351114)
Supplement: S1 File — (DOCX) [file pone.0351114.s001.docx]

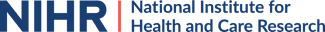


**PROSPERO**

International prospective register of systematic reviews

**Effectiveness of using immersive virtual reality as a rehabilitation intervention for people with stroke: Systematic Review.**

*Dana Islam, Maria Jaensson, Ingela Marklund, Mia Von Euler*

**Citation**

Dana Islam, Maria Jaensson, Ingela Marklund, Mia Von Euler. Effectiveness of using immersive virtual reality as a rehabilitation intervention for people with stroke: Systematic Review.. PROSPERO 2025 CRD420250639792. Available from <https://www.crd.york.ac.uk/PROSPERO/view/CRD420250639792>.

REVIEW TITLE AND BASIC DETAILS

**Review title**

Effectiveness of using immersive virtual reality as a rehabilitation intervention for people with stroke: Systematic Review.

**Review objectives**

How can immersive VR alone or in combination with other rehabilitation techniques affect limb function, balance, gait, quality of life and fatigue in people with stroke?

**Keywords**

Immersive virtual reality; Stroke; Rehabilitation

SEARCHING AND SCREENING

**Searches**

Searches will be conducted in Medline ALL (Ovid), Embase.com (Elsevier), Cochrane Library (Wiley) and CINAHL (Ebscohost) together with the librarian at Örebro University.

In Embase conference abstracts will be excluded.

**Study design**

Only randomized study types will be included.

**Link to search strategy**

A full search strategy is not available.

ELIGIBILITY CRITERIA

**Condition or domain being studied**

*Stroke Rehabilitation; Virtual Reality; Rehabilitation Therapy; Physiotherapy*

Stroke is the third most common cause of death in Sweden and the primary cause of acquired permanent disability in adults. As more individuals survive strokes and life expectancy increases, a greater number of people live with residual functional impairments after a stroke, impacting not just the individuals themselves but also society as a whole, leading to high costs in municipal and regional healthcare. Rehabilitation after a stroke is essential for improving impaired functions and activities, which helps enhance participation in society and health-related quality of life. A challenge in stroke rehabilitation is achieving adequate training intensity and frequency. Technology, including virtual reality (VR), is taking on an increasingly vital role in stroke rehabilitation. VR is categorized into non-immersive and immersive systems. Non-immersive VR is more affordable and utilizes computers or television screens. Immersive VR provides a 360-degree interactive experience in a pre-programmed environment that allows for repetition, varying intensities, and training in specific tasks using headsets, screen glasses, and sometimes even sensor-equipped gloves, enabling users to interact with virtual elements and objects. VR can boost motivation during rehabilitation by providing a variety of activities that enable individuals to engage in tasks they find appealing or even explore new experiences.

**Population**

*Included*

**Inclusion criteria:**

- 18 years and older.
- Randomized controlled trials (RCT).
- Immersive VR.

*Excluded*

**Exclusion criteria:**

- Studies not involving patients with stroke (eg multiple sclerosis, Parkinson's disease, spinal cord injury, traumatic brain injury, pain, cerebral palsy).
- Studies only using non-immersive VR.
- Studies using VR for non-rehabilitative purposes (e.g., pain management, psychological therapy).
- Studies that primarily focus on cognitive functions or outcomes without evaluating motor or functional improvements will be excluded.

**Intervention(s) or exposure(s)**

*Included*

*Virtual Reality; Rehabilitation Therapy; Physiotherapy*

This review focuses on immersive virtual reality (VR) interventions in stroke rehabilitation. Immersive VR is defined as the use of head-mounted displays or equivalent devices to create a fully immersive, interactive virtual environment designed to simulate real-world scenarios. Patients engage in task-specific exercises designed to improve motor function, balance, and gait.

**Comparator(s) or control(s)**

*Included*

*PICO tags selected: Placebo; Usual Care*

**Context**

Interventions delivered in clinical environments (e.g., hospitals, rehabilitation centers, outpatient clinics) will be included.

OUTCOMES TO BE ANALYSED

**Main outcomes**

Limb's function, Balance, Gait, Fatigue, Quality of Life and the type of instruments that are used.

**1- Motor Function Improvement**

Definition: Change in motor performance of the upper and/or lower extremities.

Measurement Instruments: Fugl-Meyer Assessment (FMA), Box and Block Test (BBT), Wolf Motor Function Test (WMFT), Action Research Arm Test (ARAT), Stroke Impact Scale (SIS).

**2- Balance**

Definition: Improvement in static and dynamic balance.

Measurement Instruments: Berg Balance Scale (BBS), Timed Up and Go (TUG), Postural Assessment Scale for Stroke Patients (PASS).

**3- Gait**

Definition: Improvement in walking ability, including speed, endurance, and quality of gait.

Measurement Instruments: 6-Minute Walk Test (6MWT), 10-Meter Walk Test (10MWT), Functional Ambulation Category (FAC).

**4- Patient-Reported Outcomes**

Definition: Patient-reported measures of motivation, fatigue, and quality of life.

Measurement Instruments:

***Motivation***: Behavioral Regulation in Exercise Questionnaire (BREQ-3).

***Fatigue:*** Fatigue Severity Scale (FSS).

***Quality of Life:*** Stroke Impact Scale (SIS), EuroQol-5D (EQ-5D), RAND-36, Stroke Specific Quality of Life Scale (SS-QOL).

**Time Points**: Pre and post intervention.

**Effect Measure:** Mean difference (MD) or standardized mean difference (SMD) with 95% confidence intervals (CIs).

**Additional outcomes**

Adverse events, compliance with the intervention, attrition, type of VR.

DATA COLLECTION PROCESS

**Data extraction (selection and coding)**

Covidence will be used, and the screening process will be carried out by a doctoral student and the supervisors. To validate the screening process, all researchers will conduct 10 title and abstract screenings together. Subsequently, we will continue the process individually, with each researcher blinded to the other's vote. Full-text screening will follow, first conducted together for the initial two articles and then performed individually. Throughout the screening process, all articles need two votes; if there are conflicting opinions, a discussion will take place, and a consensus will be reached.

Data related to the objectives will be extracted and recorded in a data extraction form created for this study. The following data will be collected from all papers included:

• Bibliographic details (lead author, title, journal, year, country of origin, full citation).

• Type of intervention and control, effects of intervention on outcomes, context/clinical setting, patients (age, gender).

• Types of measurements used.

• Timing of measurements.

• Adverse events.

• Primary outcome variables (Limb function, balance, gait, fatigue, quality of Life)

• Compliance with the intervention

• Attrition

**Risk of bias (quality) assessment**

The included studies will be assessed following the Joanna Briggs Institute checklist for RCTs.

PLANNED DATA SYNTHESIS

**Strategy for data synthesis**

- If the material permits, a meta-analysis will be conducted.
- If the data is heterogeneous, a narrative analysis will be made of the results of included articles.
- The GRADE system will be used to review reliability.

REVIEW AFFILIATION, FUNDING AND PEER REVIEW

**Review team members**

**Mrs Dana Islam**. Örebro University, Faculty of Medicine and Health, School of Health Sciences, Sweden. Sweden.

No conflict of interest declared.

**Dr Maria Jaensson** (review guarantor and contact) Örebro University, Faculty of Medicine and Health, School of Health Sciences, Sweden. Sweden.

No conflict of interest declared.

**Dr Ingela Marklund**. Centre for Clinical Research and Education, Region Värmland, Sweden. Sweden.

No conflict of interest declared.

**Professor Mia Von Euler**. Sophiahemmet University, Stockholm, Sweden. Sweden.

No conflict of interest declared.

**Named contact**

**Dr Maria Jaensson** (Maria.Jaensson@oru.se). Örebro University, Faculty of Medicine and Health, School of Health Sciences, Sweden. Sweden.

**Review affiliation**

Örebro University

**Funding source**

Review has no specific/external funding but is supported by guarantor/review team (non-commercial) institutions.

*Additional information about funding*

This study was supported by grants from the Centre for Clinical Research and Education, Region Värmland.

**Named contact**

Dana Islam. Östra Raden 365227Karlstad
dana.islam@oru.se

TIMELINE OF THE REVIEW

**Review timeline**

Start date: 1 April 2025. End date: 1 January 2026.

**Date of first submission to PROSPERO**

18 March 2025

**Date of registration in PROSPERO**

18 March 2025

CURRENT REVIEW STAGE

**Publication of review results**

Results of the review will be published in English.

**Stage of the review at this submission** 1 change

| **Review stage** | **Started** | **Completed** |
| --- | --- | --- |
| Pilot work | ✓ | ✓ |
|  |  |  |
| Formal searching/study identification | ✓ | ✓ |
|  |  |  |
| Screening search results against inclusion criteria | ✓ | ✓ |
|  |  |  |
| Data extraction or receipt of IPD | ✓ | ✓ |
|  |  |  |
| Risk of bias/quality assessment | ✓ | ✓ |
|  |  |  |
| Data synthesis | ✓ | ✓ |

**Review status**

The review is completed.

ADDITIONAL INFORMATION

**Additional information**

Further research is nedded to optimize intervention. The use of VR is a fairly new area in rehabilitation and needs more research.

**PROSPERO version history** 1 change

- Version 1.1, published 24 Jan 2026
- Version 1.0, published 18 Mar 2025

**Review conflict of interest**

Declared individual interests are recorded under team member details.. No additional interests are recorded for this review.

**Country**

Sweden

**Medical Subject Headings**

Stroke; Stroke Rehabilitation; Virtual Reality Exposure Therapy; Adult; Fatigue; Motivation; Quality of Life; Sweden

**Revision note** 1 change

Because I have finished analyzing the data

**Disclaimer**

The content of this record displays the information provided by the review team. PROSPERO does not peer review registration records or endorse their content.

PROSPERO accepts and posts the information provided in good faith; responsibility for record content rests with the review team. The guarantor for this record has affirmed that the information provided is truthful and that they understand that deliberate provision of inaccurate information may be construed as scientific misconduct.

PROSPERO does not accept any liability for the content provided in this record or for its use. Readers use the information provided in this record at their own risk.

Any enquiries about the record should be referred to the named review contact
