# Supplementary material for: Effects of immersive virtual reality on limb motor function, balance, gait and quality of life after stroke: A systematic review and meta-analysis
Source: PLoS One. 2026 Jul 6;21(7):e0351114. doi: 10.1371/journal.pone.0351114 (PMC13336215; doi:10.1371/journal.pone.0351114)
Supplement: S2 Table — Search strings used for all databases included in the systematic review. (DOCX) [file pone.0351114.s002.docx]

**Database:** Ovid MEDLINE(R) ALL 1946 to March 31, 2025

**Host:** Ovid

**Date searched:** 2025-04-01

**Limits applied:** English language

| **Field Code** | **Definition** |
| --- | --- |
| / | Mesh-term |
| exp | exploded Mesh-term |
| ab | abstract |
| kf | keyword heading word |
| ti | Title |
| pt | Publication Type |
| fs | Floating Sub-Heading |

| **Concept** | **#** | **Search** | **Results** |
| --- | --- | --- | --- |
| Stroke | 1 | exp Stroke/ or Stroke Rehabilitation/ or Hemiplegia/ or exp Gait Disorders, Neurologic/ | 211524 |
|  | 2 | (stroke* or poststroke or post-stroke or "cerebrovascular accident*" or apoplex* or "cerebral infarct*" or "subcortical infarct*" or "brain stem infarct*" or hemipares* or Hemiplegi*).ab,kf,ti. | 401141 |
|  | 3 | 1 or 2 | 442618 |
| Virtual reality | 4 | exp Virtual Reality/ | 8247 |
|  | 5 | Augmented Reality/ | 1840 |
|  | 6 | ("virtual realit*" or vr or "augmented realit*" or "immersive realit*" or "vr headset*" or "vr glasses" or "mixed realit*" or "head mounted display*" or "head mounted device*" or "immersive environment*" or "immersive virtual environment*" or "smart glasses").ab,kf,ti. | 35619 |
|  | 7 | 4 or 5 or 6 | 36756 |
| Sets combined | 8 | 3 and 7 | 1801 |
| RCT filter* | 9 | exp randomized controlled trial/ | 636608 |
|  | 10 | controlled clinical trial.pt. | 95685 |
|  | 11 | randomized.ab. | 684897 |
|  | 12 | placebo.ab. | 257067 |
|  | 13 | drug therapy.fs. | 2796426 |
|  | 14 | randomly.ab. | 456167 |
|  | 15 | trial.ab. | 744724 |
|  | 16 | groups.ab. | 2827997 |
|  | 17 | or/9-16 | 6244002 |
| Search combined with RCT filter | 18 | 8 and 17 | 773 |
| Limit to English language | 19 | limit 18 to english language | 754 |

*rows 9-16 is a modified version of the search filter *Cochrane Highly Sensitive Search Strategy for identifying randomized trials in MEDLINE: sensitivity-maximizing version (2023 revision); Ovid format(1)*. The modification that has been made is that the final two lines, designed to exclude animal studies, has been omitted.

1. Lefebvre C, Glanville J, Briscoe S, Featherstone R, Littlewood A, Metzendorf M-I, Noel-Storr A, Paynter R, Rader T, Thomas J, Wieland LS. Technical Supplement to Chapter 4: Searching for and selecting studies [last updated September 2024]. In: Higgins JPT, Thomas J, Chandler J, Cumpston M, Li T, Page MJ, Welch VA (editors). Cochrane Handbook for Systematic Reviews of Interventions version 6.5. Cochrane, 2024. Available from [www.training.cochrane.org/handbook](http://www.training.cochrane.org/handbook).

**Database**: Embase.com

**Host:** Elsevier

**Date searched:** 2025-04-01

**Limits applied:** English language, exclusion of conference abstracts

| **Field Code** | **Definition** |
| --- | --- |
| /de | Emtree term |
| /exp | exploded Entree term |
| ab, | abstract |
| kw | author keyword |
| ti, | title |
| /lim | limit |
| /it | Publication types |
| tt | a word in the original non-English title |
| it/ | publication type |

| **Concept** | **#** | **Search** | **Results** |
| --- | --- | --- | --- |
| Stroke | 1 | 'cerebrovascular accident'/exp OR 'stroke rehabilitation'/de OR 'hemiplegia'/de OR 'hemiparesis'/exp OR 'neurologic gait disorder'/exp | 544190 |
|  | 2 | stroke*:ti,ab,kw OR poststroke:ti,ab,kw OR 'post stroke':ti,ab,kw OR 'cerebrovascular accident*':ti,ab,kw OR apoplex*:ti,ab,kw OR 'cerebral infarct*':ti,ab,kw OR 'subcortical infarct*':ti,ab,kw OR 'brain stem infarct*':ti,ab,kw OR hemipares*:ti,ab,kw OR hemiplegi*:ti,ab,kw | 632777 |
|  | 3 | #1 OR #2 | 780282 |
| Virtual reality | 4 | 'virtual reality system'/exp OR 'virtual reality'/de OR 'augmented reality'/de OR 'smart glasses'/de | 37595 |
|  | 5 | 'virtual realit*':ti,ab,kw OR vr:ti,ab,kw OR 'augmented realit*':ti,ab,kw OR 'immersive realit*':ti,ab,kw OR 'vr headset*':ti,ab,kw OR 'vr glasses':ti,ab,kw OR 'mixed realit*':ti,ab,kw OR 'head mounted display*':ti,ab,kw OR 'head mounted device*':ti,ab,kw OR 'mixed reality':ti,ab,kw OR 'immersive environment*':ti,ab,kw OR 'immersive virtual environment*':ti,ab,kw OR 'smart glasses':ti,ab,kw | 44970 |
|  | 6 | #4 OR #5 | 57839 |
| Sets combined | 7 | #3 AND #6 | 2998 |
| RCT filter* | 8 | 'randomized controlled trial'/exp | 878321 |
|  | 9 | 'controlled clinical trial'/de | 445290 |
|  | 10 | random*:ti,ab,tt | 2189791 |
|  | 11 | 'randomization'/de | 100568 |
|  | 12 | 'intermethod comparison'/de | 313335 |
|  | 13 | placebo:ti,ab,tt | 392215 |
|  | 14 | compare:ti,tt OR compared:ti,tt OR comparison:ti,tt | 671228 |
|  | 15 | (evaluated:ab OR evaluate:ab OR evaluating:ab OR assessed:ab OR assess:ab) AND (compare:ab OR compared:ab OR comparing:ab OR comparison:ab) | 3108237 |
|  | 16 | (open NEXT/1 label):ti,ab,tt | 122816 |
|  | 17 | ((double OR single OR doubly OR singly) NEXT/1 (blind OR blinded OR blindly)):ti,ab,tt | 294997 |
|  | 18 | 'double blind procedure'/de | 230615 |
|  | 19 | (parallel NEXT/1 group*):ti,ab,tt | 35288 |
|  | 20 | crossover:ti,ab,tt OR 'cross over':ti,ab,tt | 133613 |
|  | 21 | ((assign* OR match OR matched OR allocation) NEAR/6 (alternate OR group OR groups OR intervention OR interventions OR patient OR patients OR subject OR subjects OR participant OR participants)):ti,ab,tt | 502688 |
|  | 22 | assigned:ti,ab,tt OR allocated:ti,ab,tt | 539199 |
|  | 23 | (controlled NEAR/8 (study OR design OR trial)):ti,ab,tt | 509779 |
|  | 24 | volunteer:ti,ab,tt OR volunteers:ti,ab,tt | 298615 |
|  | 25 | 'human experiment'/de | 687729 |
|  | 26 | trial:ti,tt | 459943 |
|  | 27 | #8 OR #9 OR #10 OR #11 OR #12 OR #13 OR #14 OR #15 OR #16 OR #17 OR #18 OR #19 OR #20 OR #21 OR #22 OR #23 OR #24 OR #25 OR #26 | 6992037 |
|  | 28 | ((random* NEXT/1 sampl* NEAR/8 ('cross section*' OR questionnaire* OR survey OR surveys OR database OR databases)):ti,ab,tt) NOT ('comparative study'/de OR 'controlled study'/de OR 'randomised controlled':ti,ab,tt OR 'randomized controlled':ti,ab,tt OR 'randomly assigned':ti,ab,tt) | 3617 |
|  | 29 | 'cross-sectional study'/de NOT ('randomized controlled trial'/exp OR 'controlled clinical trial'/de OR 'controlled study'/de OR 'randomised controlled':ti,ab,tt OR 'randomized controlled':ti,ab,tt OR 'control group':ti,ab,tt OR 'control groups':ti,ab,tt) | 434558 |
|  | 30 | 'case control*':ti,ab,tt AND random*:ti,ab,tt NOT ('randomised controlled':ti,ab,tt OR 'randomized controlled':ti,ab,tt) | 23391 |
|  | 31 | 'systematic review':ti,tt NOT (trial:ti,tt OR study:ti,tt) | 318793 |
|  | 32 | nonrandom*:ti,ab,tt NOT random*:ti,ab,tt | 20132 |
|  | 33 | 'random field*':ti,ab,tt | 3136 |
|  | 34 | ('random cluster' NEAR/4 sampl*):ti,ab,tt | 1748 |
|  | 35 | review:ab AND review:it NOT trial:ti,tt | 1271514 |
|  | 36 | 'we searched':ab AND (review:ti,tt OR review:it) | 57290 |
|  | 37 | 'update review':ab | 153 |
|  | 38 | (databases NEAR/5 searched):ab | 80487 |
|  | 39 | (rat:ti,tt OR rats:ti,tt OR mouse:ti,tt OR mice:ti,tt OR swine:ti,tt OR porcine:ti,tt OR murine:ti,tt OR sheep:ti,tt OR lambs:ti,tt OR pigs:ti,tt OR piglets:ti,tt OR rabbit:ti,tt OR rabbits:ti,tt OR cat:ti,tt OR cats:ti,tt OR dog:ti,tt OR dogs:ti,tt OR cattle:ti,tt OR bovine:ti,tt OR monkey:ti,tt OR monkeys:ti,tt OR trout:ti,tt OR marmoset*:ti,tt) AND 'animal experiment'/de | 1294550 |
|  | 40 | 'animal experiment'/de NOT ('human experiment'/de OR 'human'/de) | 2727605 |
|  | 41 | #28 OR #29 OR #30 OR #31 OR #32 OR #33 OR #34 OR #35 OR #36 OR #37 OR #38 OR #39 OR #40 | 4774710 |
|  | 42 | #27 NOT #41 | 6152919 |
| Search combined with RCT filter | 43 | #7 AND #42 | 1052 |
| Exclusion of conference abstracts | 44 | #43 NOT 'conference abstract'/it | 791 |
| Limit to English language | 45 | #43 NOT 'conference abstract'/it AND [english]/lim | 770 |

*line 8-42 has been pasted from the **Embase RCT filter for Embase.com 30 April 2023 revision** by Glanville et al <https://sites.google.com/a/york.ac.uk/issg-search-filters-resource/home/rcts/embase-rct-filter#h.33an36c3ldl>

**Database:** Cochrane Library

**Host:** Wiley

**Date searched:** 2025-04-01

**Limits applied:** none

| **Field Code** | **Definition** |
| --- | --- |
| / MeSH descriptor: [] explode all trees | exploded Mesh-term |
| MeSH descriptor: [] this term only | unexplode Mesh-term |
| ab | abstract |
| kw | keywords |
| ti | title |

| **Concept** | **#** | **Search** | **Results** |
| --- | --- | --- | --- |
| Stroke | #1 | MeSH descriptor: [Stroke] explode all trees | 17829 |
|  | #2 | MeSH descriptor: [Stroke Rehabilitation] this term only | 4138 |
|  | #3 | MeSH descriptor: [Hemiplegia] this term only | 1005 |
|  | #4 | MeSH descriptor: [Gait Disorders, Neurologic] explode all trees | 1017 |
|  | #5 | (stroke* or poststroke or post-stroke or cerebrovascular NEXT accident* or apoplex* or cerebral NEXT infarct* or subcortical NEXT infarct* or brain NEXT stem NEXT infarct* or hemipares* or Hemiplegi*):ti,ab,kw | 83829 |
|  | #6 | #1 OR #2 OR #3 OR #4 OR #5 | 84523 |
| Virtual reality | #7 | MeSH descriptor: [Virtual Reality] this term only | 1345 |
|  | #8 | MeSH descriptor: [Augmented Reality] this term only | 128 |
|  | #9 | (virtual NEXT realit* or vr or augmented NEXT realit* or immersive NEXT realit* or vr NEXT headset* or "vr glasses" or mixed NEXT realit* or head NEXT mounted NEXT display* or head NEXT mounted NEXT device* or immersive NEXT environment* or immersive NEXT virtual NEXT environment* or "smart glasses"):ti,ab,kw | 10036 |
|  | #10 | #7 OR #8 OR #9 | 10036 |
| Sets combined | #11 | #6 AND #10 | 1189 |

**Database:** CINAHL with Full Text

**Host:** EbscoHost (the old interface)

**Date searched:** 2025-04-01

**Limits applied:** English language

| **Field Code** | **Definition** |
| --- | --- |
| MH | CINAHL heading |
| MH "…+" | exploded CINAHL heading (example: MH "Stroke+") |
| TI | title |
| AB | abstract |

| **Concept** | **#** | **Search** | **Results** |
| --- | --- | --- | --- |
| Stroke | 1 | (MH "Stroke+") OR (MH "Stroke Nursing") OR (MH "Hemiplegia") OR (MH "Gait Disorders, Neurologic+") | 88454 |
|  | 2 | TI ( stroke* or poststroke or post-stroke or "cerebrovascular accident*" or apoplex* or "cerebral infarct*" or "subcortical infarct*" or "brain stem infarct*" or hemipares* or Hemiplegi* ) OR AB ( stroke* or poststroke or post-stroke or "cerebrovascular accident*" or apoplex* or "cerebral infarct*" or "subcortical infarct*" or "brain stem infarct*" or hemipares* or Hemiplegi* ) | 127497 |
|  | 3 | S1 OR S2 | 148868 |
| Virtual reality | 4 | (MH "Virtual Reality+") OR (MH "Augmented Reality") | 7880 |
|  | 5 | TI ( "virtual realit*" or vr or "augmented realit*" or "immersive realit*" or "vr headset*" or "vr glasses" or "mixed realit*" or "head mounted display*" or "head mounted device*" or "immersive environment*" or "immersive virtual environment*" or "smart glasses" ) OR AB ( "virtual realit*" or vr or "augmented realit*" or "immersive realit*" or "vr headset*" or "vr glasses" or "mixed realit*" or "head mounted display*" or "head mounted device*" or "immersive environment*" or "immersive virtual environment*" or "smart glasses" ) | 10574 |
|  | 6 | S4 OR S5 | 14102 |
| Sets combined | 7 | S3 AND S6 | 921 |
| Narrow by Language: - english | 8 | S3 AND S6 | 862 |

Note: no RCT filter was applied.
