# Supplementary material for: Effects of immersive virtual reality on limb motor function, balance, gait and quality of life after stroke: A systematic review and meta-analysis
Source: PLoS One. 2026 Jul 6;21(7):e0351114. doi: 10.1371/journal.pone.0351114 (PMC13336215; doi:10.1371/journal.pone.0351114)
Supplement: S4 Table — (DOCX) [file pone.0351114.s004.docx]

**Quality Assessment Using Joanna Briggs Institute JBI Checklist for RCTs**

|  | | | Selection and allocation | | | Administration of intervention/exposure | | | Assessment, detection and measurement of the outcome | | | Participant retention | Statistical conclusion validity | | |  |
| --- | --- | --- | --- | --- | --- | --- | --- | --- | --- | --- | --- | --- | --- | --- | --- | --- |
|  | Study | **Titel** | **Q1** | **Q2** | **Q3** | **Q4** | **Q5** | **Q6** | **Q7** | **Q8** | **Q9** | **Q10** | **Q11** | **Q12** | **Q13** | **Risk of bias** |
| 1 | **Abdollahi 2014** | Error augmentation enhancing arm recovery in individuals | Y | Y | U | Y | Y | Y | Y | N | Y | Y | Y | Y | Y | L |
| 2 | **Amin 2024** | Effectiveness of Immersive Virtual Reality-Based Hand Rehabilitation Games for Improving | Y | Y | Y | N | N | U | Y | Y | Y | Y | Y | Y | Y | H |
| 3 | **Bao 2024** | The Impact of Virtual Reality Training Combined with Traditional Chinese Medicine Health Preservation | Y | U | Y | U | N | U | Y | U | Y | Y | Y | Y | Y | H |
| 4 | **Bashir 2024** | The comparison of the caren virtual reality system-based protocol and routine physical therapy on balance | Y | U | Y | N | N | U | U | Y | Y | Y | Y | Y | Y | H |
| 5 | **Chen 2023** | Effectiveness, safety and patients' perceptions of an immersive virtual reality-based exercise system | Y | Y | Y | Y | N | Y | Y | Y | Y | Y | Y | Y | Y | L |
| 6 | **Moon 2024** | The Effects of Training with Immersive Virtual Reality Devices on Balance, Walking…. | Y | Y | N | U | N | U | Y | Y | Y | Y | Y | Y | Y | H |
| 7 | **Connelly 2010** | A pneumatic glove and immersive virtual reality environment for hand rehabilitative training after stroke | U | U | Y | N | N | U | Y | Y | Y | Y | Y | Y | Y | H |
| 8 | **Crosbie 2012** | Virtual reality in the rehabilitation of the upper limb after hemiplegic stroke: a randomised pilot study | Y | Y | Y | N | N | Y | Y | Y | Y | Y | Y | Y | Y | L |
| 9 | **Dąbrowská 2023** | Effect of Virtual Reality Therapy on Quality of Life and Self-Sufficiency in Post-Stroke Patients | Y | U | Y | N | N | U | Y | Y | Y | Y | Y | Y | Y | H |
| 10 | **Hegazy**  **2022** | Impact of a virtual reality program on post-stroke upper limb function: a randomized controlled trial | Y | Y | Y | Y | N | Y | Y | Y | Y | Y | Y | Y | Y | L |
| 11 | **Hsu 2022** | Effects of a Virtual Reality-Based Mirror Therapy Program on Improving Sensorimotor Function of Hands in Chronic Stroke | Y | Y | Y | N | N | Y | Y | Y | Y | Y | Y | Y | Y | L |
| 12 | **Huang 2022** | Effects of virtual reality-based motor control training on inflammation…. | Y | Y | Y | N | N | Y | Y | Y | Y | Y | Y | Y | Y | L |
| 13 | **Huang 2024** | Immersive virtual reality‑based rehabilitation for subacute stroke: a randomized controlled trial | Y | Y | Y | N | N | Y | Y | Y | Y | Y | Y | Y | Y | L |
| 14 | **Jo 2024** | 360° immersive virtual reality-based mirror therapy for upper extremity function and satisfaction | Y | Y | Y | N | N | Y | Y | Y | Y | Y | Y | Y | Y | L |
| 15 | **Jung 2012** | Effects of Virtual Reality Treadmill Training on Balance and Balance Self-efficacy in Stroke Patients…… | Y | Y | Y | N | N | Y | Y | Y | Y | Y | Y | Y | Y | L |
| 16 | **Kim 2009** | Use of Virtual Reality to EnhanceBalance and Ambulation in Chronic Stroke | Y | U | Y | Y | N | Y | Y | Y | Y | Y | Y | Y | Y | L |
| 17 | **Kuo 2023** | Effects of a wearable sensor–based virtual reality game on upper-extremity  function in patients with stroke | Y | Y | Y | N | N | Y | Y | Y | Y | Y | Y | Y | Y | L |
| 18 | **Kwak 2024** | The effect of balance training using touch controller-based fully immersive virtual reality devices on balance and walking…. | Y | Y | Y | U | U | U | Y | U | Y | Y | Y | Y | Y | H |
| 19 | **Kwon 2012** | Effects of virtual reality on upper extremityfunction and activities of daily livingperformance in acute strok | Y | U | Y | Y | N | Y | Y | U | Y | Y | Y | Y | Y | L |
| 20 | **Lee CH 2014** | Augmented reality-based postural control  training improves gait function in patients  with stroke | Y | Y | Y | N | N | Y | Y | Y | Y | Y | Y | Y | Y | L |
| 21 | **Lee SJ 2014** | Combination Transcranial Direct Current Stimulation and Virtual Reality Therapy for Upper Extremity  Training in Patients with Subacute Stroke | Y | Y | Y | N | Y | Y | Y | Y | Y | Y | Y | Y | Y | L |
| 22 | **Loganathan 2024** | Effectiveness of reinforced feedback in virtual environment for upper limb rehabilitation in acute stroke | Y | U | Y | N | N | U | Y | N | Y | Y | Y | Y | Y | H |
| 23 | **Marda 2023** | Effect of task-oriented balance training versus virtual reality-based balance training in stroke patients-a comparative study | Y | U | U | N | N | N | Y | Y | Y | Y | Y | Y | Y | H |
| 24 | **Mekbib 2021** | A novel fully immersive virtual reality environment for upper extremity rehabilitation in patients with stroke | Y | Y | Y | N | N | Y | Y | U | Y | Y | Y | Y | Y | L |
| 25 | **ÖGÜN 2019** | Effect of leap motion-based 3D immersive virtual reality usage on upper extremity | Y | Y | Y | Y | N | Y | Y | Y | Y | Y | Y | Y | Y | L |
| 26 | **Park 2013** | Clinical usefulness of the virtual reality-based postural control | Y | U | Y | N | N | U | Y | Y | Y | Y | Y | Y | Y | H |
| 27 | **Peláez-Vélez 2023** | Use of Virtual Reality and Videogames in the Physiotherapy Treatment of Stroke Patients | Y | Y | Y | N | N | Y | Y | Y | Y | Y | Y | Y | Y | L |
| 28 | **Shaphe 2018** | Efficacy of closed loop feedback system with augmented virtual reality visual cues training on gait and functional | Y | Y | Y | N | N | N | Y | Y | Y | Y | Y | Y | Y | H |
| 29 | **Shin 2015** | Effects of game-based virtual reality on health-related quality of life | Y | Y | Y | N | N | Y | Y | Y | Y | Y | Y | Y | Y | L |
| 30 | **Sip 2023** | Perspectives of Motor Functional Upper Extremity Recovery with the Use of Immersive Virtual Reality | Y | U | U | N | N | N | N | U | Y | Y | Y | Y | Y | H |
| 31 | **Song et al 2021** | 2021 Effect of Immersive Virtual Reality-Based Bilateral Arm Training in Patients with Chronic Stroke | Y | Y | Y | N | N | N | Y | Y | Y | Y | Y | Y | Y | H |
| 32 | **Subramanian 2013** | Arm Motor Recovery Using a Virtual Reality Intervention in Chronic Stroke | Y | Y | Y | N | Y | Y | Y | N | Y | Y | Y | Y | Y | L |
| 33 | **Yang 2008** | Virtual reality-based training improves community ambulation in individuals | Y | Y | Y | N | N | Y | Y | Y | Y | Y | Y | Y | Y | L |
| 34 | **Zakharov 2020** | Stroke Affected Lower LimbsRehabilitation Combining Virtual Reality With Tactile Feedback | Y | U | N | N | N | Y | Y | Y | Y | Y | Y | Y | Y | H |

*Y = Yes, N = No, U = Unclear*

*L: low risk of bias*

*H: High risk of bias*

1- Was true randomization used for assignment of participants to treatment groups? Yes, no, unclear.

2. Was allocation to groups concealed? Yes, no, unclear.

3. Were treatment groups similar at the baseline? Yes, no, unclear.

4. Were participants blind to treatment assignment? Yes, no, unclear.

5. Were those delivering treatment blind to treatment assignment? Yes, no, unclear.

6. Were outcomes assessors blind to treatment assignment? Yes, no, unclear.

7. Were treatment groups treated identically other than the intervention of interest? Yes, no, unclear.

8. Was follow up complete and if not, were differences between groups in terms of their follow-up adequately described and analyzed? Yes, no, unclear.

9. Were participants analyzed in the groups to which they were randomized? Yes, no, unclear.

10. Were outcomes measured in the same way for treatment groups? Yes, no, unclear.

11. Were outcomes measured in a reliable way? Yes, no, unclear.

12. Was appropriate statistical analysis used? Yes, no, unclear.

13. Was the trial design appropriate for the topic, and any deviations from the standard RCT design accounted for in the conduct and analysis? Yes, no, unclear.
